# Age-structured malaria model with temperature-dependent dynamics and optimal control analysis within a partial differential equation framework

**DOI:** 10.1371/journal.pone.0330158

**Published:** 2025-08-20

**Authors:** Lukas Degu Petros, Temesgen Erena Abdisa, Dinka Tilahun Etefa, Dawit Kechine Menbiko, Ademe Kebede Gizaw, Eba Alemayehu Simma, Delenasaw Yewhalaw, Chernet Tuge Deressa

**Affiliations:** 1 Department of Mathematics, Jimma University, Jimma, Ethiopia; 2 Department of Biology, Jimma University, Jimma, Ethiopia; 3 School of Medical Laboratory Sciences, Institute of Health, Jimma University, Jimma, Ethiopia; University of Dhaka, BANGLADESH

## Abstract

Malaria remains a significant global health challenge, particularly in sub-Saharan Africa, despite advances in control measures. In 2023, there were an estimated 263 million malaria cases and 597,000 deaths, with most occurring in Africa. This study presents a temperature-dependent, two-class age-structured malaria model using partial differential equations and optimal control strategies to assess their impact on malaria transmission. We analyze the existence and stability of equilibria, determined by the basic reproduction number *R*_0_, and demonstrate global stability through Lyapunov functionals. Numerical simulations show the effects of temperature variations and optimal controls on transmission dynamics, providing actionable insights for malaria management. Empirical validation of the model was performed using six years of infection prevalence data from the Jimma zone, revealing an ${\text{R}}^{2}$ of 0.68 and an adjusted ${\text{R}}^{2}$ of 0.63, indicating a good fit to observed data. Furthermore, comparison with an existing age-structured malaria model from the literature showed superior predictive accuracy, with our model demonstrating better performance in capturing temperature-dependent malaria trends. These results underscore the robustness and practical relevance of the model, offering improved prediction and control strategies under varying environmental conditions.

## 1 Introduction

Malaria is one of the oldest and most destructive infectious diseases affecting humans. This life-threatening, vector-borne illness is caused by the *Plasmodium* parasite and is transmitted through the bite of an infected female Anopheles mosquito [1]. In spite of significant measures taken to prevent, control, and eliminate malaria, it continues to pose significant health challenges, specially in tropical regions, including parts of Africa, Asia, and the Americas, which provide favorable conditions for its rapid spread [2]. If left untreated, infected individuals may suffer severe complications and even fatal outcomes. *Anopheles* mosquitoes, especially those carrying *Plasmodium* falciparum, transmit the disease to humans while feeding on blood.

Unlike humans, mosquitoes do not govern their basal temperatures [3]. Both the female Anopheles mosquito vector and *Plasmodium* malaria parasites have biological clocks that are highly sensitive to temperature changes [4]. Additionally, the aquatic habitats of immature *Anopheles* mosquitoes are greatly affected by rainfall and local hydrodynamic conditions. Variations in climatic factors can create environments that are conducive to the development and reproduction of both the malaria parasite and its mosquito vector. This could potentially lead to the emergence of malaria in formerly disinfected areas or alter the intensity of transmission through shifts in mosquito biting patterns driven by seasonal changes [5]. In the African tropics, the high prevalence of malaria is largely attributed to environmental conditions that favor mosquito larval development and the maturation of the parasite within the infected mosquito [6–8]. Temperature plays a critical role in the life cycles of both the *Anopheles* vector and the *Plasmodium* parasite. Numerous studies have shown that mosquito activity increases in warmer temperatures [3,9–11]. Rainfall creates breeding sites for mosquitoes, thereby expanding their habitats [8–12]. As a result, temperature and rainfall significantly impact malaria transmission dynamics. However, these are not the only influencing factors; seasonality, geography, host age, and other environmental factors also influence the transmission dynamics of malaria.

Temperature between $14{\;}^{\circ }\text{C}-18{\;}^{\circ }\text{C}$ and $35{\;}^{\circ }\text{C}-40{\;}^{\circ }\text{C}$ significantly impact vector-borne diseases transmission [7]. Temperatures in the range of $14{\;}^{\circ }\text{C}$ to $18{\;}^{\circ }\text{C}$ are expected to increase the frequency of malaria transmission events, making transmission more persistent or recurring in areas where it was previously sporadic [13]. For malaria, temperature-influences *anopheles* mosquitoes frequency (increasing temperature due to faster blood digestion) and larval development (accelerated maturation in warmer water) [7,13,14]. Adult female mosquito lifespan(normally about 21 days) decreases rapidly above $\left[30{\;}^{\circ }\text{C},\;32{\;}^{\circ }\text{C}\right]$ [15]. Temperature equally affects the parasite development within the mosquito: maturation takes 19 days at $22{\;}^{\circ }\text{C}$, but eight days at $30{\;}^{\circ }\text{C}$ [15,16]. Temperatures exceeding $34{\;}^{\circ }\text{C}$ negatively impact vector and parasite survival, reducing transmission [17]. With rising global temperatures, vector distribution may shift, potentially increasing malaria incidence in cooler endemic areas and decreasing it in regions exceeding optimal temperatures (provided other factors remain constant) [18,19].

Beck-Johnson *et al*. in [20] developed a temperature-dependent, stage-structured delayed differential equation model to investigate the impact of climate on mosquito borne disease risk. The model, which incorporates the full mosquito life cycle, reveals that the mosquito population abundance is more sensitive to temperature than previously thought because it is strongly influenced by the dynamics of the juvenile mosquito stages whose vital rates are also temperature-dependent.

Agusto *et al*. in [21] examined the influence of temperature fluctuations on malaria transmission and shown, using mean monthly temperature data from 67 cities across the four sub-Saharan regions of Africa, and showed that malaria burden (measured in terms of the total number of new cases of infection) increases with increasing temperature between $16{\;}^{\circ }\text{C}-28{\;}^{\circ }\text{C}$ and decreases for temperature values above $28{\;}^{\circ }\text{C}$ in West Africa, $27{\;}^{\circ }\text{C}$ in Central Africa, $26{\;}^{\circ }\text{C}$ in East Africa, and $25{\;}^{\circ }\text{C}$ in South Africa. Again Agusto *et al*. in [22] also demonstrated the total number of new malaria cases rises as the mean monthly temperature increases from $19.04{\;}^{\circ }\text{C}-26.75{\;}^{\circ }\text{C}$ in East African cities (Kigali, Rwanda, Gulu, Uganda, Lodwar and Kenya). They also demonstrated that personal protection, particularly the use of bed nets, should be encouraged not only at low temperatures but particularly at high temperatures when individuals avoid the use of bed nets. Furthermore, control and reduction of malaria may be possible even when mosquitoes develop resistance to insecticides.

Wang *et al*. in [23], developed a model to explore the dynamics of malaria using a class an age-structure to examine the influences of age of vaccine, age of recovery, and relapse. By extending the model into an optimal control framework, it was concluded that spraying of insecticide at the time of an epidemic is more efficient than protective actions alone for disease control. To get ride of malaria, they suggest implementing a range of preventive strategies early in the epidemic, such as immunization through vaccine, the use of insecticide-treated nets, and other protective measures. Besides, they demonstrated that combining these preventive efforts with insecticide spraying remarkably enhances effectiveness of disease control.

In [24], a compartmental vector host model, SEIR-LSEI is formulated to asses the impact of temperature in the transmission dynamics and incidence of malaria disease by considering temperature dependent parameters. The numerical results showed that the temperature levels lower than $16{\;}^{\circ }\text{C}$ and greater than $32{\;}^{\circ }\text{C}$ have negative impact on the survival of the mosquitoes and the transmission dynamics of malaria. Therefore, peoples living in this range of temperature are at a lower risk of malaria. Meanwhile, they showed that large population get infected of malaria when the temperature is moderate. Thus, moderate temperature is advantageous for the survival of the mosquitoes and transmission of the disease as well.

Numerous studies have examined malaria transmission and burden in relation to seasonal factors such as temperature and various control mechanisms. Despite the efforts, malaria remains a serious public health threat. This persistent challenge underscores the need for further investigation to deepen our understanding of the infection and reduce its impact. Therefore, this study aims to develop a new two-class age-structured, temperature-dependent malaria model that incorporates the immature stage of mosquitoes with the framework of partial differential equations which has not been addressed in the existing literature. Additionally, the model introduces three optimal control strategies to assess the effects of temperature and these control measures on the transmission dynamics of malaria.

The paper is organized as follows: Sect 2 presents the formulation of the model. Detailed descriptions of the temperature-dependent parameters are provided in Sect 3. In Sect 4, the existence of solution and stability of the equilibria is discussed. The model is extended to include an optimal control problem in Sect 5. Numerical results and discussions are presented in Sects 6 and 7, separately.

## 2 Model formulation

Compartmental malaria model with temperature dependent parameters was followed to study the effect of temperature on malaria. To formulate the model, total human population: *N*_*h*_ is categorized into susceptible group: *S*_*h*_, vaccinated group: *V*, the infected group: *I*_*h*_ and the recovered group: *R*. Thus,


$$N_{h}(t)=S_{h}(t)+V(a,\;t)+I_{h}(t)+R(b,\;t),$$

where, V(a,t) is the density of the vaccinated humans at time *t* and vaccination age *a*, with the total number of vaccinated individuals within the vaccinated subclass at time *t* being $\int_{0}^{\infty }V(a,\;t)da$. R(b,t) is the density of the recovered individuals at time *t* with recovery age *b*, and hence $\int_{0}^{\infty }R(b,\;t)db$ is the total number of recovered humans in the recovered class at time *t*.

Since temperature affects both immature and mature stages of mosquitoes, we divide the vectors into two stages: immature stage and adult stage. Hence, the total mosquito population at time t: *N*_*m*_(*t*) is subdivided into *L*(*t*): immature stages of mosquito, susceptible mosquito: *S*_*m*_(*t*), exposed mosquitoes: *E*_*m*_(*t*), and infected mosquitoes: *I*_*m*_(*t*). That is


$$N_{m}(t)=L(t)+S_{m}(t)+E_{m}(t)+I_{m}(t).$$

We assume that the rate at which vaccine-induced immunity fades depends on the age at which the vaccination is delivered and is expressed as ξ(a), thus $\int_{0}^{\infty }\xi (a)V(a,\;t)da$ is the total number of wanning of immunity that progress into the susceptible class. In the same manner, we assume that γ(b) is the age-dependent relapse rate of individuals in the recovery class. Therefore, $\int_{0}^{\infty }\gamma (b)R(b,\;t)db$ is the total number of humans who enter into the infectious class from the recovery class. Based on the descriptions of state variables, the SVIR-LSEI model with two class-age structures, temperature dependent parameters and relapse will take the following form.

$$\left\{ \begin{array}{c} \frac{dS_{h}}{dt}=\Lambda_{h}-\psi_{h}S_{h}-\mu_{h}S_{h}-\frac{\lambda_{mh}bI_{m}S_{h}}{N_{h}}+\int_{0}^{\infty }\xi (a)V(a,\;t)\;da,\\ V_{a}+V_{t}=-\xi (a)V(a,\;t)-\mu_{h}V(a,\;t),\\ V(0,t)=\psi_{h}S_{h},\\ \frac{dI_{h}}{dt}=\frac{\lambda_{mh}bI_{m}S_{h}}{N_{h}}-\beta_{1}I_{h}-\mu_{h}I_{h}-\nu I_{h}+\int_{0}^{\infty }r(b)R(b,\;t)\;db,\\ R_{b}+R_{t}=-\mu_{h}R(b,\;t)-r(b)R(b,\;t),\\ R(0,t)=\beta_{1}I_{h},\\ \frac{dL}{dt}=\phi \left(1-\frac{L}{K}\right)(S_{m}+E_{m}+I_{m})-\omega_{l}L-\sigma_{m}L,\\ \frac{dS_{m}}{dt}=\sigma_{m}L-\omega S_{m}-\frac{\lambda_{hm}bI_{h}S_{m}}{N_{h}},\\ \frac{dE_{m}}{dt}=\frac{\lambda_{hm}bI_{h}S_{m}}{N_{h}}-\omega E_{m}-\beta_{2}E_{m},\\ \frac{dI_{m}}{dt}=\beta_{2}E_{m}-\omega I_{m}, \end{array}\right.$$ (1)

with initial conditions; $S_{h}(0)=S_{h0},\;I_{h}(0)=I_{h0},\;S_{m}(0)=S_{m0},\;E_{m}(0)=E_{m0},\;I_{m}(0)=I_{m0}.$

To ensure the well-posedness of the age-structured partial differential equations in our model, we explicitly specify the initial and boundary conditions for the vaccinated and recovered compartments.

- For the vaccinated population *V*(*a*,*t*), governed by the PDE
  $$V_{a}+V_{t}=-\xi (a)V(a,\;t)-\mu_{h}V(a,\;t),$$
- we impose the boundary condition at age *a* = 0 as
  $$V(0,t)=\psi_{h}S_{h}(t),$$
- which models the inflow of newly vaccinated individuals from the susceptible class. The initial age distribution of vaccinated individuals is given by
  $$V(a,0)=V_{0}(a),\;a\in [0,\infty ),\;\text{with }V_{0}(a)\in L_{+}^{1}({\mathbb{R}}_{+}).$$
- For the recovered population *R*(*b*,*t*), described by
  $$R_{b}+R_{t}=-\mu_{h}R(b,\;t)-r(b)R(b,\;t),$$
- we specify the boundary condition
  $$R(0,t)=\beta_{1}I_{h}(t),$$
- representing the flow of recovered individuals from the infected class. The initial condition is given by
  $$R(b,0)=R_{0}(b),\;b\in [0,\infty ),\;\text{with }R_{0}(b)\in L_{+}^{1}({\mathbb{R}}_{+}).$$

**Fig 1:**
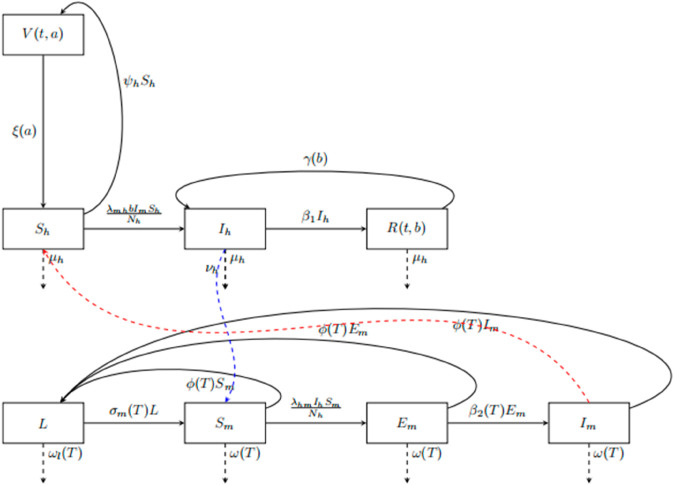
Flow diagram representing the transmission dynamics between human and vector populations.

The temperature dependent parameters are $\sigma_{m}=\sigma_{m}(T),\;\beta_{1}=\beta_{1}(T),\;\beta_{2}=\beta_{2}(T),\;\omega_{l}=\omega_{l}(T),\;\omega =\omega (T),\;b=b(T)$ and ϕ=ϕ(T). According to the state variables and parameters described in Tables 1 and 2 respectively, the flow chart for the model (1) is given in Fig 1.

**Table 1:** State variables and their descriptions.

variables	descriptions
*S*_*h*_(*t*)	Total number of susceptible humans
*V*(*a*,*t*)	Total number of vaccinated human with age of vaccination a
*I*_*h*_(*t*)	Total number of infectious humans
*R*(*b*,*t*)	Total number of recovered human with age of recovery b
*L*(*t*)	Total number of immature vectors
*S*_*m*_(*t*)	Total number of susceptible mosquito vectors
*E*_*m*_(*t*)	Total number of exposed vectors
*I*_*m*_(*t*)	Total number of infectious vectors

**Table 2:** Parameters and their biological interpretations of model (1).

parameters	their interpretations
$\wedge_{h}$	recruitment rates of humans
$\psi_{h}$	the vaccination rate of the susceptible humans
$\mu_{h}$	death rate of humans
$\lambda_{mh}$	probability of human infection from mosquito bite
$\lambda_{hm}$	probability of transmission from humans to mosquitoes
$\beta_{1}$	recovery rates of humans
ν	diseases induced death rates of humans
*ϕ*	temperature dependent Egg deposition rate from each adult mosquito compartment
$\omega_{l}$	death rate of immature mosquitoes which is temperature dependent
$\sigma_{m}$	transmission rate from immature to adult mosquitoes which is temperature dependent
*ω*	adult mosquito death rate which is temperature dependent
$\beta_{2}$	transmission rate from exposed to infectious mosquitoes

**Assumptions**

- In our model, the age-structured variables depend on:
  a∈[0,∞): the time since vaccination for individuals in the vaccinated class *V*(*a*,*t*), and b∈[0,∞): the time since recovery for individuals in the recovered class *R*(*b*,*t*).
  The partial differential equations governing *V*(*a*,*t*) and *R*(*b*,*t*) are defined on the semi-infinite domains a,b∈[0,∞).
- For $a\geq 0,\;b\geq 0:\;S_{h0},\;I_{h0},\;S_{m0},\;I_{m0},\;E_{m0}$ and *L*_0_ are in *R*  and $V_{0}(a),\;R_{0}(b)$ are in *R*  and bounded almost everywhere integrable functions in the Lebesgue sense.
- $\sigma_{m}(T),\;\beta_{1}(T),\;\beta_{2}(T),\;\omega_{l}(T),\;\omega (T),\;b(T),\;and\;\phi (T)$ are nonnegative temperature dependent functions. $\lambda_{mh}$ and $\lambda_{hm}$ are the probabilities of human infection from mosquito bite and probability of transmission from humans to mosquitoes respectively.
- $\mu_{h},\;\psi_{h},\;\nu$ are assumed to be nonnegative constants.
- Functions which are dependent on ages; ω(a),γ(b) meet the stated assumptions below, provided that $R_{+}=[0,\;\infty ]$ and $L_{+}^{\infty }$ are the set of Lebesgue integrable functions which are essentially bounded and positive.
  (*H*_1_). $\bar{\omega },\;\bar{\gamma }$ are upper bounds of ω(a)andγ(b), respectively which are in $L_{+}^{\infty }(0,\;\infty )$.
  (*H*_2_). $M_{\omega },\;M_{\gamma }$ respectively are Lipischitzians of ω(a),γ(b) which are Lipschitz continuous and defined in *R*_ + _.
  (*H*_3_). There exists a constant $\mu_{0}\in (0,\;\mu_{h}]$ such that $\omega (\tau ),\;\gamma (\tau )\geq \mu_{0},\;\forall \tau >0$.

To analyze the dynamics of model (1), let the state variable at time *t* be defined as:


$$U(t):=\left(S_{h}(t),I_{h}(t),L(t),S_{m}(t),E_{m}(t),I_{m}(t),V(\cdot ,t),R(\cdot ,t)\right).$$

We define the Banach space


$$X:={\mathbb{R}}_{+}^{6}\times L_{+}^{1}(0,\infty )\times L_{+}^{1}(0,\infty ),$$

equipped with the norm


$$\|U(t){\|}_{X}:=|S_{h}(t)|+|I_{h}(t)|+|L(t)|+|S_{m}(t)|+|E_{m}(t)|+|I_{m}(t)|+\|V(\cdot ,\;t){\|}_{L^{1}}+\|R(\cdot ,\;t){\|}_{L^{1}}.$$

We seek solutions U(t)∈X for all t≥0, and assume that


U∈C([0,T];X),

meaning each component is continuous in time, and the age-structured functions *V* and *R* are integrable in *a* and *b* respectively, for every t∈[0,T], and the initial conditions of the model (1) is;


$$X_{0}=(S_{h0},\;V_{0}(a),\;I_{h0},\;R_{0}(b),\;L_{0},\;S_{m0},\;E_{m0},\;I_{m0})\in X;\;\;V(0,\;0)=\psi_{h}S_{h0}\;and\;R(0,\;0)=\beta_{1}I_{h0}.$$

According to Wang in [23], uniqueness, existence, non-negativity and continuous solutions of the model are guaranteed. Hence, the model has a non-negative and single solution;


$$S(0,t)=(S_{h}(t),\;V(\cdot ,\;t),\;I_{h}(t),\;R(\cdot ,\;t),\;L(t),\;S_{m}(t),\;E_{m}(t),\;I_{m}(t)),\;\forall t\geq 0;$$

and initial conditions $S(0,\;X_{0})=X_{0}\in X$, with

$$||S(t,x_{0})|{|}_{X}=|S_{h}(t)|+\int_{0}^{\infty }|V(a,t)|da\;+|I_{h}(t)|+\int_{0}^{\infty }|R(b,t)|db+|L(t)|+|S_{m}(t)|+|E_{m}(t)|+|I_{m}(t)|.$$ (2)

To simplify notations, for a,b≥0 assume that


$$\xi_{1}(a)=\mu_{h}+\xi (a),\;\xi_{2}(b)=\mu_{h}+\gamma (b),\;\eta_{1}=\int_{0}^{\infty }\xi (a)k_{1}(a)da,$$

$$k_{1}(a)=e^{\int_{0}^{a}\xi_{1}(s)ds},\;k_{2}(b)=e^{\int_{0}^{b}\xi_{2}(s)ds},\;\eta_{2}=\int_{0}^{\infty }\gamma (b)k_{2}(b)db.$$

By restricting the models to partial differential equations (PDEs) and solving them along the characteristic curves defined by *t*−*a* = *constant*, following the approach in [25], we obtain

$$V(a,\;t)=\left\{ \begin{array}{c} V(t-a,\;0)e^{-\int_{0}^{a}\xi_{1}(s)ds}=\psi_{h}S_{h}(t-a)k_{1}(a),\;t>a\geq 0,\\ V_{0}(a-t)e^{-\int_{a-t}^{a}\xi_{2}(s)ds}=V_{h0}(a-t)\frac{k_{1}(a)}{k_{1}(a-t)},\;a\geq t\geq 0. \end{array}\right.$$ (3)

Similarly R(b,t) can be obtained as;

$$R(b,\;t)=\left\{ \begin{array}{c} R(t-b,0)e^{-\int_{0}^{b}\xi_{2}(s)ds}=\beta_{1}I_{h}(t-b)k_{2}(b),\;t>b\geq 0,\\ R_{0}(b-t)e^{-\int_{b-t}^{b}\xi_{2}(s)ds}=R_{0}(b-t)\frac{k_{2}(b)}{k_{(}b-t)},\;b\geq t\geq 0. \end{array}\right.$$ (4)

For the model (1), we can state the space as;


$$\Pi =\{(S_{h},\;V(\cdot ,\;t),\;I_{h},\;R(\cdot ,\;t),\;L,\;S_{m},\;E_{m},\;I_{m})\in X:S_{h}+\int_{0}^{\infty }V(a,\;t)da\;+I_{h}+\int_{0}^{\infty }R(b,\;t)db\leq \frac{\wedge_{h}}{\mu_{h}},L+S_{m}+E_{m}+I_{m}\leq K\}.$$

which is a forward invariant set, that can be shown by the next proposition.

**Proposition 2.1** *For the proposed model* (1) *above, the subsequent statements are true.*

*i.* Π *is forward invariant for S, which implies* $S(x_{0},t)\in \Pi$, *for all non-negative real numbers t and x*_0_ *is in* Π;
*ii.* *S is eventually bounded and* Π *attracts all points in X*.

*Proof*: Let $S(x_{0},\;t)=S_{1}(x_{0},\;t)+S_{2}(x_{0},\;t)$, where,


$$S_{1}(x_{0},\;t)=(S_{h}(t),\;V(\cdot ,\;t),\;I_{h}(t),\;R(\cdot ,\;t),\;0,\;0,\;0,\;0),\;\;and\;S_{2}(x_{0},\;t)\;=0,\;0,\;0,\;0,\;L,\;S_{m}(t),\;E_{m}(t),\;I_{m}(t).$$

So we get;


$$\frac{d}{dt}||S_{1}(x_{0},\;t)|{|}_{X}=\frac{dS_{h}}{dt}+\frac{\partial }{\partial t}\int_{0}^{\infty }V(a,\;t)da+\frac{dI_{h}}{dt}+\frac{\partial }{\partial t}\int_{0}^{\infty }R(b,\;t)db=\wedge_{h}-\psi_{h}S_{h}\;-\mu_{h}S_{h}-\frac{\lambda_{mh}bI_{m}}{N_{h}}S_{h}+\int_{0}^{\infty }\omega (a)V(a,\;t)da-\int_{0}^{\infty }\frac{\partial }{\partial t}V(a,\;t)da-\int_{0}^{\infty }(\mu_{h}+\omega (a))V(a,\;t)da\;+\frac{\lambda_{mh}bI_{m}}{N_{h}}S_{h}-(\beta_{1}+\mu_{h}+\nu )I_{h}+\int_{0}^{\infty }\gamma (b)R(b,\;t)db-\int_{0}^{\infty }\frac{\partial }{\partial t}R(b,\;t)db\;-\int_{0}^{\infty }(\mu_{h}+\gamma (b))R(b,\;t)db\leq \wedge_{h}-\mu_{h}(S_{h}(t)+\int_{0}^{\infty }V(a,\;t)da+I_{h}(t)+\int_{0}^{\infty }R(b,\;t)db)$$

$$⟹{lim sup}_{t\to \infty }||S_{1}(x_{0},\;t)||\leq \frac{\wedge_{h}}{\mu_{h}}.\;Moreover,\;||S_{2}(x_{0},\;t)||\leq K.\;⟹\forall t\geq 0,\;S_{1}(x_{0},\;0),\;S_{2}(x_{0},\;0)\in \Pi ,$$

for all solutions of the above model with *x*_0_ in Π. Hence, *S*(*x*_0_,*t*) is forward invariant. In addition, $S(x_{0},\;t)$ is eventually bounded and Π attracts all points which are in *X*. This completes the proof. □

## Practical implementation and future direction

While the current model incorporates age structure and temperature-dependent parameters to make accurately reflect malaria transmission dynamics, we aknowledge the importance of developing tools that are accessible to public health practitioners. The complexity of our model is deliberate and biologically justified, as it captures nuanced heterogenuities essential for understanding malaria epidemiologically under climate and demographic variation.

Nonetheless, this framework can serve as a foundation for deriving simplified or reduced form models suitable for practical applications. For example, one could aggregate age groups or approximate temperature effects using fitted seasonal forcing functions. Such simplifications, while useful for rapid decision-making, may compromise biological realism and model fidelity-particularily in regions where age and temperature-specific transmission patterns are crutial. We envision future work extending this model into a family of approximate tools that preserve core mechanisms but are tailored for specific operational contexts. Additionally, the full model can inform parameter ranges and intervention strategies even when simplified models are deployed in the field.

## 3 Temperature dependent parameters

Malaria, a globally prevalent infectious disease, is increasingly impacting new populations worldwide. Temperature significantly influences the vectorial capacity of mosquitoes, thereby affecting the transmission dynamics of malaria. Studies demonstrate that temperature extremes below $16{\;}^{\circ }\text{C}$ and above $32{\;}^{\circ }\text{C}$ negatively impact the survival of both immature and adult mosquitoes, as well as the disease’s transmission [24]. Consequently, populations living in these temperature ranges face a lower risk of malaria. Conversely, mild temperatures are conducive to mosquito survival and disease transmission, leading to higher infection rates in such conditions. Furthermore, slight increases in temperature can significantly impact malaria cases in several African countries [26]. Blanford *et al*. [27] modeled the effects of daily temperature variations and mean temperature on the extrinsic incubation period of the malaria parasite. Similarly, Mordecai *et al*. [28], using a nonlinear thermal response model, found that the optimal temperature for malaria transmission is around $25{\;}^{\circ }\text{C}$, with a marked decline in transmission above $28{\;}^{\circ }\text{C}$. Agusto *et al*. [29], employing a mechanistic repeated exposure malaria model that explicitly includes the immature stages of the mosquito vector, identified a favorable temperature range for mosquito growth between $16{\;}^{\circ }\text{C}$ and $28{\;}^{\circ }\text{C}$ across 67 sub-Saharan African cities. These findings underscore the critical role of temperature in shaping malaria transmission patterns and mosquito development. Consequently, the objective of the current study is to extend the work in [23] by incorporating temperature-dependent parameters, compartments like immature stages of the mosquito and exposed mosquitoes. The temperature-dependent parameters of the model (1) are defined as follows. It should be noted that all the temperature-dependent parameters of the model (1) are positive for temperature values between $T\in [15{\;}^{\circ }\text{C},\;32{\;}^{\circ }\text{C}]$((the temperature range for the place where the experiment made at, province of south west of Ethiopia, to be used in the numerical simulation of the model). Hence, for temperatures in this range, the temperature-dependent parameters of the model (1) are well-defined.

To analyze the effect of temperature on the parameters of model (1) and the corresponding transmission dynamics of malaria, real data was obtained from the Tropical and Infectious Diseases Research Center (TIDRC), Jimma University Ethiopia [30]. The study was conducted from September 2023 to April 2024 at the Tropical and Infectious Diseases Research Centre,Jimma University in Sekoru; Oromia region ($7.922305^{0}\text{N}$, $37.395320^{0}\text{E}$). The research center is 246 km southwest of Addis Ababa, situated at an altitude of 1684 m above sea level. The area has typically one dry season (November to March) and two rainy seasons: a long rainy season from June to September, with the peak rainfall in July and August, and a short rainy season from April to May. The area receives 1940.5 mm mean annual rainfall, having heavy rains from June to August and short rains from September to December. The mean annual temperature is $13{\;}^{\circ }\text{C}$ to $20{\;}^{\circ }\text{C}$. Sekoru District experiences the coldest and hottest temperatures in August and February respectively. Rearing of An.arabiensis mosquito carried out both under variable temperatures, relative humidity and standard insectary conditions. The study involved rearing mosquitoes in simulated temperatures and relative humidity. A total of four hundred (400) eggs of An. arabiensis were obtained from JU TIDRC insectary and reared to adults.

Mosquitoes were reared across temperatures ranging from the minimum $14.55{\;}^{\circ }\text{C}$ to maximum $34.35{\;}^{\circ }\text{C}$ and relative humidity spanning from 64 to 86%. Each temperature and humidity combination were replicated four times, with one hundred mosquito eggs placed in petri dishes containing wet filter paper. After a 24-hour period, the eggs were carefully washed and transferred into hatching trays. Throughout the larval stage, adequate nourishment was provided, and observations were recorded until pupation occurred.

Subsequently, pupae were relocated into cages, and the emergence of adult mosquitoes was closely monitored until the end of their lifespan. Detailed records were maintained, capturing key parameters such as the number of days required for egg hatching, as well as the survival or mortality rates of eggs hatching, larvae, pupae and adult mosquitoes under each temperature and humidity condition. Therefore, the accuracy of the data was ensured. For fitting the collected data to the assumed temperature-dependent parameters such as the number of larvae and pupae developed, maturation rate of immature mosquitoes, and mortality rates of both immature and adult mosquitoes, a polynomial regression function model was selected. This choice was made after evaluating various curve-fitting models using Python software and hence we choose quadratic polynomials because they exhibit good empirical fit (in terms of ${\text{R}}^{2}$ and residuals) over the observed temperature range and provide a simple and flexible functional form for numerical simulation and theoretical analysis. As a sample the analysis and explanations for number of days spent as an egg stage is presented below.

**Fig 2:**
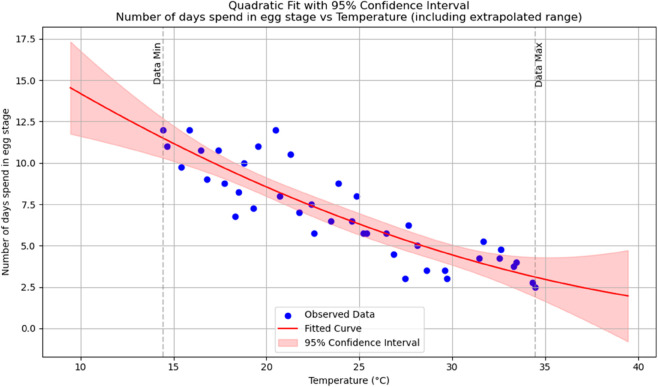
The predicted number of days spent in an egg stage as a function of temperature with a polynomial Regression function of degree two.

As it is shown in Table 3 and Fig 2, the dependent variable in the regression analysis is number of days spent at egg stage which is predicted using the independent variable (predictor) Temperature. How well the independent variable, Temperature, explains the variation in the number of days spent at egg stage is indicated by R-squared and Adjusted R-squared. The R-squared is 0.785 indicating that approximately 78.5% of the variation in the number of days is explained by Temperature. A more accurate measure of the models goodness of fit is verified by the Adjusted R-square and is 77.4%. The F-statistic of 69.56, coupled with a very small p-value of $1.99{\text{e}}^{-13}$, indicates that the regression model is highly statistically significant. Regression equation representing the number of days spent at egg status, as a function of temperature is given by the equation, $N_{0}(T)=21.3635-0.7939T+0.0077T^{2}$.

**Table 3:**
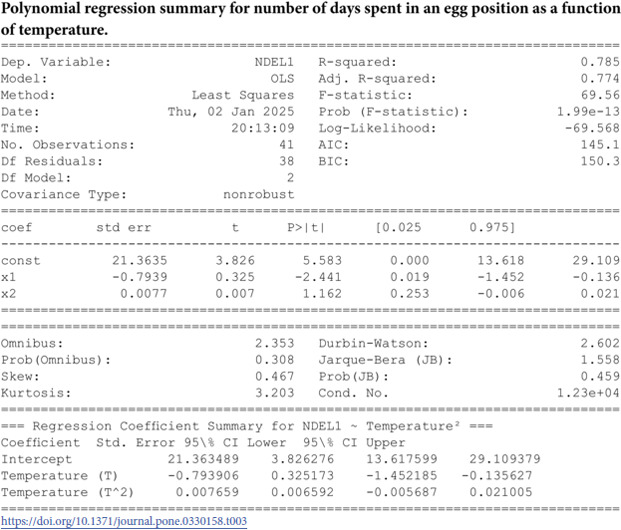
Polynomial regression summary for number of days spent in an egg position as a function of temperature.

From Fig 2, the confidence band appropriately widens beyond the data, indicating increased uncertainty in extrapolated regions.

Summary of temperature-based fitted equations for model parameters with associated goodness-of-fit metrics is shown in Table 4 and reports of statistical indicators for each model are presented in Tables [5–13] which are provided in Appendix 1 7 and their corresponding polynomial fits are also shown in Figs 3–11.

**Fig 3:**
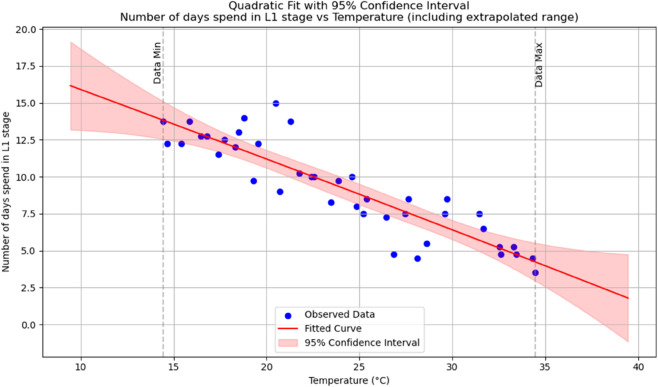
The predicted number of days spent in an *L*_1_ stage as a function of temperature with a polynomial regression function of degree two.

**Fig 4:**
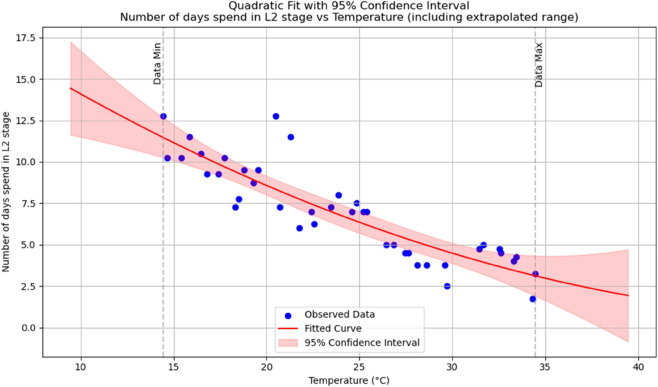
The predicted number of days spent in an *L*_2_ stage as a function of temperature with a polynomial regression function of degree two.

**Fig 5:**
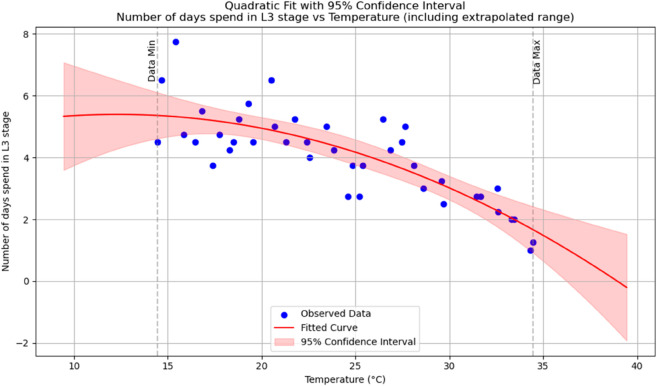
The predicted number of days spent in an *L*_3_ stage as a function of temperature with a polynomial regression function of degree two.

**Fig 6:**
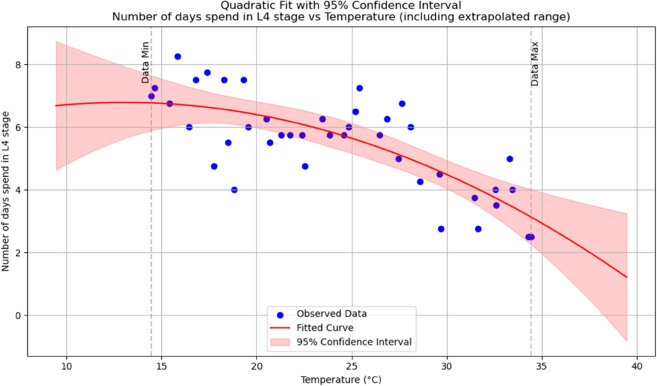
The predicted number of days spent in an *L*_4_ stage as a function of temperature with a polynomial regression function of degree two.

**Fig 7:**
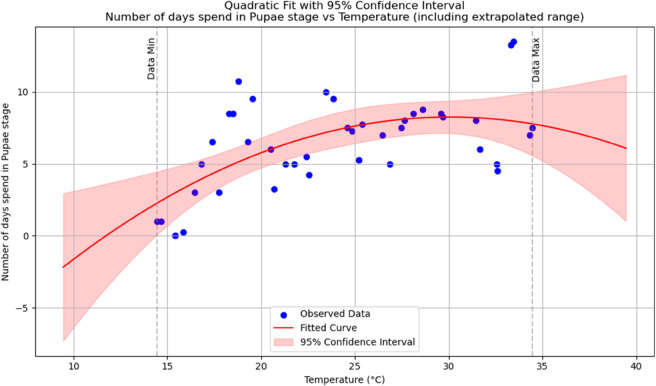
The predicted number of days spent in Pupal stage as a function of temperature with a polynomial regression function of degree two.

**Fig 8:**
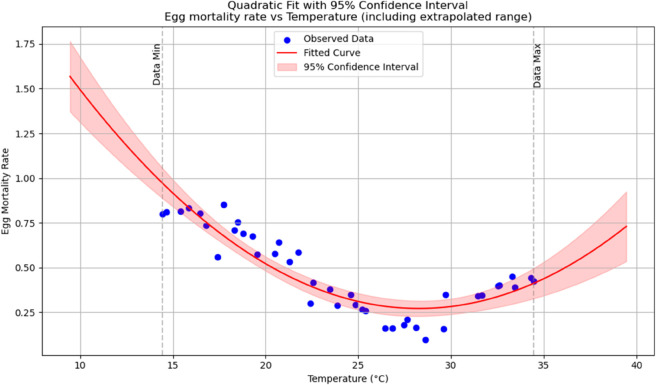
Egg mortality rate as a function of temperature with a polynomial regression function of degree two.

**Fig 9:**
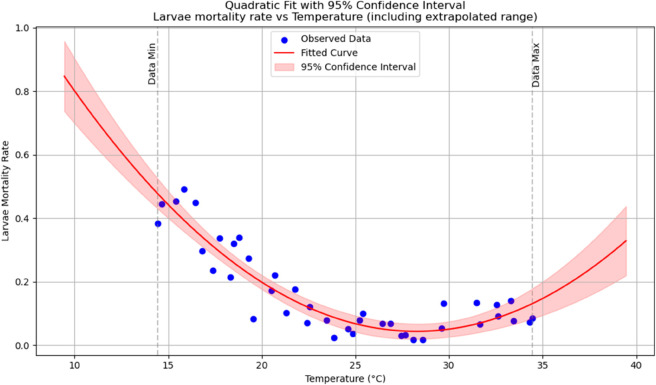
Larvae mortality rate as a function of temperature with a polynomial regression function of degree two.

**Fig 10:**
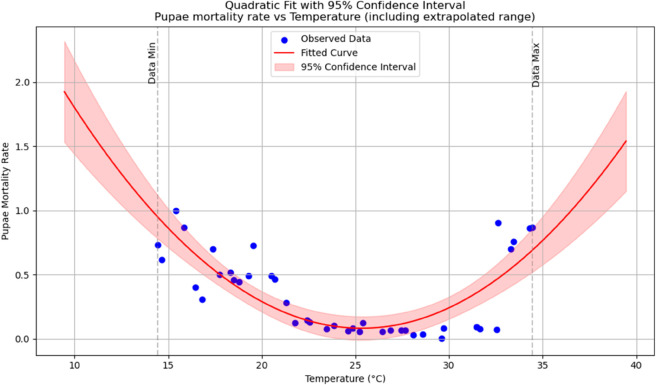
Pupae mortality rate as a function of temperature with a polynomial regression function of degree two.

**Fig 11:**
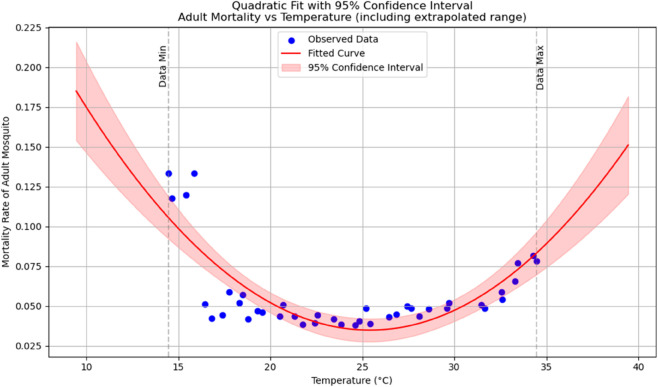
Adult mortality rate as a function of temperature with a polynomial regression function of degree two.

**Table 4:** Summary of temperature-based fitted equations for model parameters with associated goodness-of-fit metrics.

parameters	Respective Equations	R-squared	Adj.R-squared	F-statistic	P-value
*N*_0_(*T*)	21.3635−0.7939*T* + 0.0077*T*^2^	0.785	0.774	69.56	$1.99{\text{e}}^{-13}$
*N*_1_(*T*)	20.5216–0.4570*T*–$0.0005{\text{T}}^{2}$	0.806	0.796	79.14	$2.83{\text{e}}^{-14}$
*N*_2_(*T*)	21.0398−0.7664*T* + 0.0071*T*^2^	0.781	0.769	67.63	$3.02{\text{e}}^{-13}$
*N*_3_(*T*)	4.2448 + 0.1873*T*−0.0076*T*^2^.	0.658	0.640	36.48	$1.44{\text{e}}^{-09}$
*N*_4_(*T*)	5.4217 + 0.2087*T*−0.0080*T*^2^	0.576	0.553	25.77	$8.47{\text{e}}^{-08}$
*N*_5_(*T*)	5.4217 + 0.2087*T*−0.0080*T*^2^	0.361	0.327	10.71	0.000205
$\omega_{1}(T)$	3.1971−0.2070*T* + 0.0037*T*^2^	0.827	0.818	90.80	$3.35{\text{e}}^{-15}$
$\omega_{2}(T)$	1.858−0.1285*T* + 0.0023*T*^2^	0.854	0.846	110.7	$1.41{\text{e}}^{-16}$
$\omega_{3}(T)$	4.7700−0.3702*T* + 0.0073*T*^2^	0.640	0.621	33.78	$3.71{\text{e}}^{-09}$
ω(T)	0.4154−0.03*T* + 0.0006*T*^2^	0.654	0.636	35.93	$1.74{\text{e}}^{-09}$

where, $N_{0}(T),\;N_{1}(T),\;N_{2}(T),\;N_{3}(T),\;N_{4}(T)$ and *N*_5_(*T*), are number of days spent in egg, $L_{1},\;L_{2},\;L_{3},\;L_{4},$ and pupae stages respectively and $\omega_{1}(T),\;\omega_{2}(T),\;\omega_{3}(T),\;and\;\omega (T)$ are mortality rates of egg, Larva, pupa, and adult mosquito respectively.

The total time spent from egg to adult state is $N(T)=N_{0}(T)+N_{1}(T)+N_{2}(T)+N_{3}(T)+N_{4}(T)+N_{5}(T)=58.6469-0.1449T-0.0259T^{2}$. Therefore, maturation rate, $\sigma_{m}(T)=\frac{1}{N(T)}=\frac{1}{58.6469-0.1449T-0.0259T^{2}}$.

Temperature dependent mortality rate of immature mosquitoes, is given by;


$$\omega_{l}(T)=\frac{\omega_{1}N_{0}(T)+\omega_{2}(N_{1}(T)+N_{2}(T)+N_{3}(T)+N_{4}(T))+\omega_{3}N_{5}(T)}{N(T)}\;\;\;\;\;\;\;\;\;\;\;\;\;\;\;\;\;\;\;\;\;\;\;\;\;\;\;\;\;$$

$$=\frac{-0.00017718T^{4}+0.01516252T^{3}-0.30523159T^{2}-2.87583464T+96.96741905}{58.6469-0.1449T-0.0259T^{2}}.$$

Mordecai *et al*. in [11] experimentally suggested that the biting rate as a function of T, *b*(*T*) defined by $b(T)=\left(\frac{203T^{2}}{10^{6}}-\frac{2375.1T}{10^{6}}\right)\sqrt{32.4-T}$. We can take the rate of egg deposition ϕ(T) as $\phi (T)=-97.7+8.61T-0.153T^{2}$ from [7].

## 4 Equilibria and their stability

By assuming that $(S_{h}^{*}$, $V^{*}(\cdot ),I_{h}^{*},R^{*}(\cdot ),L^{*},S_{m}^{*},E_{m}^{*},I_{m}^{*})$ is an equilibrium point of model (1), it must satisfy the equation:




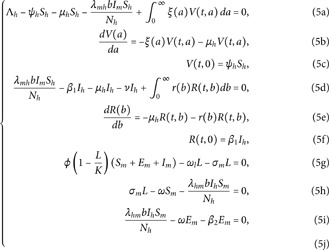




We can easily see that Eq (5) has a disease free equilibrium point $\left(S_{h0},V_{0}(a),0,0,L_{0},S_{m0},0,0\right)$, where,


$$S_{h0}=\frac{\wedge_{h}}{\mu_{h}+\psi_{h}(1-\eta_{1})},\;V_{0}(a)=\psi_{h}S_{h0}k_{1}(a),\;S_{m0}=\frac{\sigma_{m}K}{\omega }-\frac{K}{\phi }(\omega_{l}+\sigma_{m})\;\;and\;L_{0}=K-\frac{K\omega }{\phi \sigma_{m}}(\omega_{l}+\sigma_{m})$$

### 4.1 Basic reproduction number and existence of equilibria

The basic reproduction number denoted by *R*_0_ is the number of secondary infections from a single infective individual on its entire infectious period. Based on the values of the number, disease can sustain if *R*_0_>1 and the disease dies out if *R*_0_<1. Hence, we compute *R*_0_, associated with the disease free equilibrium point as follows.

There are $\frac{\wedge_{h}}{\mu_{h}+\psi_{h}(1-\eta_{1})}$ susceptible people and $\frac{K}{\omega }$ susceptible female anopheles mosquitoes. A primary infectious human case a removal rate $\beta_{1}+\mu_{h}+\nu$ and the average infectious period is $\frac{1}{\mu_{h}+\nu +\beta_{1}(1-\eta_{2})}$. During this time the average number of mosquito bites from the susceptible mosquito is $\frac{b}{\mu_{h}+\nu +\beta_{1}(1-\eta_{2})}$. So, the average number of infected but not infectious mosquitoes from the infectious human case will be $\frac{b}{\mu_{h}+\nu +\beta_{1}(1-\eta_{2})}\frac{K}{\omega }\lambda_{hm}$, then $\frac{bK\lambda_{hm}}{(\beta_{1}(1-\eta_{2})+\mu_{h}+\nu )\omega }$ represents the total number of infectious female anopheles mosquitoes produced by infected but not infectious mosquitoes. The infectious mosquitoes have a removal rate $\omega +\beta_{2}$, the average infectious period is $\frac{1}{\omega +\beta_{2}}$. During this time, the average number of mosquito bites from the susceptible mosquitoes is $\frac{b}{\omega +\beta_{2}}$, so that the average number of infected but not infectious human cases from the infectious mosquitoes will be;


$$\frac{\wedge_{h}bK\lambda_{hm}\lambda_{mh}\beta_{2}b}{(\beta_{1}(1-\eta_{2})+\mu_{h}+\nu )\omega (\omega +\beta_{2})(\mu_{h}+\psi_{h}(1-\eta_{1}))N_{h}^{2}}.$$

Therefore, $R_{0}=\frac{\wedge_{h}b^{2}K\lambda_{hm}\lambda_{mh}\beta_{2}}{(\beta_{1}(1-\eta_{2})+\mu_{h}+\nu )\omega (\omega +\beta_{2})(\mu_{h}+\psi_{h}(1-\eta_{1}))N_{h}^{2}}S_{m}^{*},\;S_{m}^{*}=\frac{\sigma_{m}K}{\omega }\;-\;\frac{K}{\phi }(\omega_{l}+\sigma_{m})$. To find an endemic equilibrium, if exists set $\left(S_{h}^{*},\;V^{*}(\cdot ),\;I_{h}^{*},\;R^{*}(\cdot ),\;L^{*},\;S_{m}^{*},\;E_{m}^{*},\;I_{m}^{*}\right)$ is an endemic equilibrium, then it must satisfy Eq (5). Now from Eqs 5b and 5e respectively we have;

$$V^{*}(a,t)=\psi_{h}S_{h}^{*}k_{1}(a)\;\;and\;\;R^{*}(b)=\beta_{1}I_{h}^{*}k_{2}(b).$$ (6)

Similarly from 5a and 5i respectively we have;

$$S_{h}^{*}=\frac{\wedge_{h}}{\mu_{h}+\frac{\lambda_{mh}bI_{m}^{*}}{N_{h}}+\psi_{h}(1-\eta_{1})}\;\;and\;\;E_{m}^{*}=\frac{\omega I_{m}^{*}}{\beta_{2}}.$$ (7)

From Eq 5d, we have;

$$I_{h}^{*}=\frac{\wedge_{h}\frac{\lambda_{mh}bI_{m}^{*}}{N_{h}}}{[\mu_{h}+\nu +\beta_{1}(1-\eta_{2})]\left[\mu_{h}+\frac{\lambda_{mh}bI_{m}^{*}}{N_{h}}+\psi_{h}(1-\eta_{1})\right]}.$$ (8)

Again from 5i and Eq (7), we get;

$$S_{m}^{*}=\frac{\omega N_{h}I_{m}^{*}(\omega +\beta_{2})}{\lambda_{hm}bI_{h}^{*}\beta_{2}}.$$ (9)

Now from Eq 5h we have;

$$L^{*}=\frac{\omega +\frac{\lambda_{hm}bI_{h}^{*}}{N_{h}}}{\sigma_{m}}$$ (10)

Inserting Eqs (6) – (10) into Eq (5i) we get;


$$MHI(I_{m}^{*}{)}^{4}+(MHC+MIJ)(I_{m}^{*}{)}^{3}+(MIC+A)(I_{m}^{*}{)}^{2}+BI_{m}^{*}-M\phi =0,\;where$$

$$D=\frac{\lambda_{mh}b}{N_{h}},\;G=\frac{\lambda_{hm}b}{N_{h}},\;E=\lambda_{hm}b\beta_{2},\;F=\omega^{2}N_{h}+\omega N_{h}+\omega N_{h}\beta_{2},\;P=\mu_{h}+\nu +\beta_{1}(1-\eta_{2}),$$

$$Q=\mu_{h}+D+\psi_{h}(1-\eta_{1}),\;C=\frac{\omega +\beta_{2}}{\beta_{2}},\;X=\frac{\phi \omega }{K\sigma_{m}},\;Z=\frac{G\phi }{K\sigma_{m}},\;Y=\frac{F}{G\beta_{2}N_{h}},\;R=\omega_{l}+\sigma_{m},$$

$$H=\frac{PQXY}{D\wedge_{h}},\;I=YZ,\;J=\frac{PQF}{ED\wedge_{h}},\;J=PQR\omega F,\;B=GD\wedge_{h}RF,\;and\;M=\sigma_{m}GN_{h}\beta_{2}D\wedge_{h}.$$

If we put $f(I_{m}^{*})=MHI{\left(I_{m}^{*}\right)}^{4}+(MHC+MIJ){\left(I_{m}^{*}\right)}^{3}+(MIC+A){\left(I_{m}^{*}\right)}^{2}+BI_{m}^{*}-M\phi =0$, then $f(0)=-M\phi <0,\;f`(I_{m}^{*})>0$ and $f(K)=R_{0}\;-\;1>0,\;for\;R_{0}>1$. Therefore, the equation $f(I_{m}^{*})=0$ has a unique positive root between (0,*K*). Hence, endemic equilibrium exists for *R*_0_>1. Further, we can generalize the existence of equilibria for the model (1) using the theorem below.

**Theorem 4.1.** *Disease free equilibrium* $\left(S_{h0},\;V_{0}(a),\;0,\;0,\;L_{0},\;S_{m0},\;0,\;0\right)$ *always exists for model* (1) *and endemic equilibrium* $\left(S_{h}^{*},\;V^{*}(\cdot ),\;I_{h}^{*},\;R^{*}(\cdot ),\;L^{*},\;S_{m}^{*},\;E_{m}^{*},\;I_{m}^{*}\right)$ *exists for R*_0_>1.

### 4.2 Local stabilities of equilibria

To check local stabilities of the disease free equilibrium and endemic equilibrium, let us linearize model (1) at the generic equilibrium, $\left(S_{h}^{*},\;V^{*}(\cdot ),\;I_{h}^{*},\;R^{*}(\cdot ),\;L^{*},\;S_{m}^{*},\;E_{m}^{*},\;I_{m}^{*}\right)$. That is let $S_{h}^{*}(t)=S_{h}^{*}\;+\;x_{1}(t)$, $V(a,t)=V^{*}(a)\;+\;x_{2}(a,t),\;I_{h}^{*}(t)=I_{h}^{*},\;R(b,t)=R^{*}(b)\;+\;x_{4}(b,t),\;L(t)=L^{*}\;+\;x_{5}(t),\;S_{m}^{*}(t)=S_{m}^{*}\;+\;x_{6}(t),\;E_{m}^{*}(t)=E_{m}^{*}\;+\;x_{7}(t),\;I_{m}^{*}(t)=I_{m}^{*}\;+\;x_{8}(t)$, where $x_{1}(t),\;x_{2}(a,t),$ $x_{3}(t),\;\cdots x_{8}(t)$ are the perturbations of the equilibrium.

Plugging these terms into the model (1), rearranging and dropping the non-linear terms we get the system;

$$\left\{ \begin{array}{c} \frac{dx_{1}}{dt}=-\left(\mu_{h}+\psi_{h}+\frac{\lambda_{mh}bI_{m}^{*}}{N_{h}}\right)x_{1}(t)-\frac{\lambda_{mh}b}{N_{h}}S_{h}^{*}x_{8}(t)+\int_{0}^{\infty }\xi (a)x_{2}(a,t)\;da,\\ \frac{\partial x_{2}}{\partial a}+\frac{\partial x_{2}}{\partial t}=-\xi (a)x_{2}(a,t)-\mu_{h}x_{2}(a,t),\;x_{2}(0,t)=\psi_{h}x_{1}(t),\\ \frac{dx_{3}}{dt}=\frac{\lambda_{mh}bI_{m}^{*}x_{1}(t)}{N_{h}}+\frac{\lambda_{mh}b}{N_{h}}x_{8}(t)S_{h}^{*}-\left(\beta_{1}+\mu_{h}+\nu \right)x_{3}(t)+\int_{0}^{\infty }r(b)x_{4}(b,t)\;db,\\ \frac{\partial x_{4}}{\partial b}+\frac{\partial x_{4}}{\partial t}=-\mu_{h}x_{4}(b,t)-r(b)x_{4}(b,t),\;x_{4}(0,t)=\beta_{1}x_{3}(t),\\ \frac{dx_{5}}{dt}=\phi \left(1-\frac{L^{*}}{K}\right)(x_{6}(t)+x_{7}(t)+x_{8}(t))-\frac{\phi x_{5}(t)}{K}\left(S_{m}^{*}+E_{m}^{*}+I_{m}^{*}\right)-(\omega_{l}+\sigma_{m})x_{5}(t),\\ \frac{dx_{6}}{dt}=\sigma_{m}x_{5}(t)-\omega x_{6}(t)-\frac{\lambda_{hm}bI_{h}^{*}x_{6}(t)}{N_{h}}-\frac{\lambda_{hm}bx_{3}(t)S_{m}^{*}}{N_{h}},\\ \frac{dx_{7}}{dt}=\frac{\lambda_{hm}bI_{h}^{*}x_{6}(t)}{N_{h}}+\frac{\lambda_{hm}bx_{3}(t)S_{m}^{*}}{N_{h}}-(\omega +\beta_{2})x_{7}(t),\\ \frac{dx_{8}}{dt}=\beta_{2}x_{7}(t)-\omega x_{8}(t). \end{array}\right.$$ (11)

We find the solution of system (11) as an exponential form by letting, $x_{1}(t)=\overset{―}{x_{1}}e^{\lambda t},\;x_{2}(a,t)=\overset{―}{x_{2}(a)}e^{\lambda t}$, $x_{3}(t)=\overset{―}{x_{3}}e^{\lambda t},\;x_{4}(b,t)=\overset{―}{x_{4}(b)}e^{\lambda t},\;x_{5}(t)=\overset{―}{x_{5}}e^{\lambda t},\;x_{6}(t)=\overset{―}{x_{6}}e^{\lambda t},\;x_{7}(t)=\overset{―}{x_{7}}e^{\lambda t},\;x_{8}(t)=\overset{―}{x_{8}}e^{\lambda t}$.

Therefore inserting these values into the system (11), rearranging and evaluate at the diseases free equilibrium we get;

$$\left\{ \begin{array}{c} \left(\mu_{h}+\nu +\lambda +\beta_{1}(1-\eta_{2})\right)\bar{x_{3}}-\frac{\lambda_{mh}bS_{h}^{*}\bar{x_{8}}}{N_{h}}=0,\\ -\left(\frac{\phi }{K}S_{m}^{*}+\omega_{l}+\lambda +\sigma_{m}\right)\bar{x_{5}}+\phi \left(1-\frac{L^{*}}{K}(\bar{x_{6}}+\bar{x_{7}}+\bar{x_{8}})\right)=0,\\ \left(\lambda +\mu_{h}+\psi_{h}(1-\eta_{1})\right)\bar{x_{1}}+\frac{\lambda_{mh}bS_{h}^{*}}{N_{h}}\bar{x_{8}}=0,\\ \frac{\lambda_{hm}b}{N_{h}}S_{m}^{*}\bar{x_{3}}-\sigma_{m}\bar{x_{5}}+(\lambda +\omega )\bar{x_{6}}=0,\\ -\frac{\lambda_{hm}b}{N_{h}}S_{m}^{*}\bar{x_{3}}+(\lambda +\omega +\beta_{2})\bar{x_{7}}=0,\\ -\beta_{2}\bar{x_{7}}+(\lambda +\omega )\bar{x_{8}}=0. \end{array}\right.$$ (12)

Solving system (12), by going through some steps we get an expression of the form:


$$\frac{\lambda_{mh}\lambda_{hm}b^{2}\beta_{2}\wedge_{h}\left[\sigma_{m}-\frac{K}{\omega }-\frac{K}{\phi }(\omega_{l}+\sigma_{m})\right]}{N_{h}^{2}\left[\mu_{h}+\nu +\lambda +\beta_{1}(1-\eta_{2})\right](\lambda +\omega +\beta_{2})(\lambda +\omega )(\mu_{h}+\psi_{h}(1-\eta_{1}))}=1$$

Denoting this expression in 𝔾, we get;


$$𝔾(\lambda )=\frac{\lambda_{mh}\lambda_{hm}b^{2}\beta_{2}\wedge_{h}\left[\sigma_{m}-\frac{K}{\omega }-\frac{K}{\phi }(\omega_{l}+\sigma_{m})\right]}{N_{h}^{2}\left[\mu_{h}+\nu +\lambda +\beta_{1}(1-\eta_{2})\right](\lambda +\omega +\beta_{2})(\lambda +\omega )(\mu_{h}+\psi_{h}(1-\eta_{1}))}=1.$$

Clearly $𝔾(0)=R_{0},\;{𝔾}^{\prime }(\lambda )<0$. Hence, 𝔾(λ) is decreasing function of *λ*. Again $lim_{\lambda \to \infty }𝔾(\lambda )=0$. If *R*_0_>1, then G(λ)=1 has a unique positive real root. This implies the disease free equilibrium is unstable for *R*_0_>1. But if *R*_0_<1, it is locally stable. If not, $𝔾(\lambda_{0})=1$ has at least one complex root, $\lambda_{0}=a_{1}+ib_{1}$, with $a_{1}\geq 0$. But


$$|𝔾(\lambda_{0})|\leq 𝔾(\lambda )=\frac{\lambda_{mh}\lambda_{hm}b^{2}\beta_{2}\wedge_{h}\left[\sigma_{m}-\frac{K}{\omega }-\frac{K}{\phi }(\omega_{l}+\sigma_{m})\right]}{N_{h}^{2}\left[\mu_{h}+\nu +\lambda +\beta_{1}(1-\eta_{2})\right](\lambda +\omega +\beta_{2})(\lambda +\omega )(\mu_{h}+\psi_{h}(1-\eta_{1}))}=R_{0}<1.$$

Which is contradiction. Therefore, for *R*_0_<1 all roots of G(λ)=1 have negative real parts. Therefore, the disease free equilibrium is locally asymptotically stable. At the endemic equilibrium the system will be

$$\left\{ \begin{array}{c} \left(\lambda +\mu_{h}+\psi_{h}+\frac{\lambda_{mh}bI_{m}^{*}}{N_{h}}\right)\bar{x_{1}}+\frac{\lambda_{mh}bS_{h}^{*}}{N_{h}}\bar{x_{8}}-\int_{0}^{\infty }\xi (a)\bar{x_{2}}(0)k_{1}(a)e^{-\lambda a}da=0,\\ \left(\lambda +\mu_{h}+\psi_{h}(1-\eta_{1})+\frac{\lambda_{mh}bI_{m}^{*}}{N_{h}}\right)\bar{x1}+\frac{\lambda_{mh}bS_{h}^{*}}{N_{h}}\bar{x_{8}}=0,\\ \left(\mu_{h}+\nu +\lambda +\beta_{1}(1-\eta_{2})\right)\bar{x_{3}}-\frac{\lambda_{mh}bI_{m}^{*}}{N_{h}}\bar{x_{1}}-\frac{\lambda_{mh}bS_{h}^{*}}{N_{h}}\bar{x_{8}}=0,\\ \phi \left(1-\frac{L^{*}}{K}\right)(\bar{x_{6}}+\bar{x_{7}}+\bar{x_{8}})-\left[\frac{\phi }{K}\left(S_{m}^{*}+E_{m}^{*}+I_{m}^{*}\right)+\lambda +\omega_{l}+\sigma_{m}\right]\bar{x_{5}}=0,\\ \frac{\lambda_{hm}bS_{m}^{*}}{N_{h}}\bar{x_{3}}-\sigma_{m}\bar{x_{5}}+\left(\lambda +\omega +\frac{\lambda_{hm}bI_{h}^{*}}{N_{h}}\right)\bar{x_{6}}=0,\\ -\frac{\lambda_{hm}bS_{m}^{*}}{N_{h}}\bar{x_{3}}-\frac{\lambda_{hm}bI_{h}^{*}}{N_{h}}\bar{x_{6}}+(\lambda +\omega +\beta_{2})\bar{x_{7}}=0,\\ \beta_{2}\bar{x_{7}}+(\lambda +\omega )\bar{x_{8}}=0. \end{array}\right.$$ (13)

Solving system (13) and re-arranging we get an equation of the form


$$\frac{\beta_{2}\lambda_{hm}\lambda b^{3}\phi \left(1-\frac{L^{*}}{K}\right)S_{m}^{*}\sigma_{m}\lambda_{mh}^{2}I_{m}^{*}S_{h}^{*}+\left(\lambda +\mu_{h}+\frac{\lambda \lambda_{mh}bI_{m}^{*}}{N_{h}}+\psi_{h}(1-\eta_{1})\right)(\lambda_{hm}^{2}\lambda_{mh}b^{2}S_{m}^{*}S_{h}^{*}\beta_{2})}{\left(\begin{array}{c} \beta_{2}\lambda_{hm}\lambda_{mh}b^{2}I_{h}^{*}S_{h}^{*}S_{m}^{*}N_{h}\sigma_{m}\left(\lambda +\mu_{h}+\frac{\lambda \lambda_{mh}bI_{m}^{*}}{N_{h}}+\psi_{h}(1-\eta_{1})\right)\phi \left(1-\frac{L^{*}}{K}\right)\\ +\sigma_{m}N_{h}^{2}\left(\lambda +\mu_{h}+\frac{\lambda \lambda_{mh}bI_{m}^{*}}{N_{h}}+\psi_{h}(1-\eta_{1})\right)\\ (\mu_{h}+\nu +\lambda +\beta_{1}(1-\eta_{2}))N_{h}^{2}(\lambda +\omega +\beta_{2})(\lambda +\omega )\phi \left(1-\frac{L^{*}}{K}\right)\\ +\sigma_{m}N_{h}^{2}\left(\lambda +\mu_{h}+\frac{\lambda \lambda_{mh}bI_{m}^{*}}{N_{h}}+\psi_{h}(1-\eta_{1})\right)(\mu_{h}+\nu +\lambda +\beta_{1}(1-\eta_{2}))\lambda_{hm}bI_{h}^{*}\phi \left(1-\frac{L^{*}}{K}\right) \end{array}\right)}=1$$

By letting this equation by 𝔾, then


$$𝔾=\frac{\beta_{2}\lambda_{hm}\lambda b^{3}\phi \left(1-\frac{L^{*}}{K}\right)S_{m}^{*}\sigma_{m}\lambda_{mh}^{2}I_{m}^{*}S_{h}^{*}+\left(\lambda +\mu_{h}+\frac{\lambda \lambda_{mh}bI_{m}^{*}}{N_{h}}+\psi_{h}(1-\eta_{1})\right)(\lambda_{hm}^{2}\lambda_{mh}b^{2}S_{m}^{*}S_{h}^{*}\beta_{2})}{\left(\begin{array}{c} \beta_{2}\lambda_{hm}\lambda_{mh}b^{2}I_{h}^{*}S_{h}^{*}S_{m}^{*}N_{h}\sigma_{m}\left(\lambda +\mu_{h}+\frac{\lambda \lambda_{mh}bI_{m}^{*}}{N_{h}}+\psi_{h}(1-\eta_{1})\right)\phi \left(1-\frac{L^{*}}{K}\right)\\ +\sigma_{m}N_{h}^{2}\left(\lambda +\mu_{h}+\frac{\lambda \lambda_{mh}bI_{m}^{*}}{N_{h}}+\psi_{h}(1-\eta_{1})\right)\\ (\mu_{h}+\nu +\lambda +\beta_{1}(1-\eta_{2}))N_{h}^{2}(\lambda +\omega +\beta_{2})(\lambda +\omega )\phi \left(1-\frac{L^{*}}{K}\right)\\ +\sigma_{m}N_{h}^{2}\left(\lambda +\mu_{h}+\frac{\lambda \lambda_{mh}bI_{m}^{*}}{N_{h}}+\psi_{h}(1-\eta_{1})\right)(\mu_{h}+\nu +\lambda +\beta_{1}(1-\eta_{2}))\lambda_{hm}bI_{h}^{*}\phi \left(1-\frac{L^{*}}{K}\right) \end{array}\right)}=1$$

Inserting the values of $S_{h}^{*},\;S_{m}^{*},\;I_{h}^{*},\;E_{m}^{*}$ and *L*  into 𝔾, hence we get $𝔾(0)=R_{0}$. We can also see easily that ${𝔾}^{\prime }(\lambda )>0$ and $lim_{\lambda \to \infty }𝔾(\lambda )=\infty$ that indicates us 𝔾 is monotonically increasing function of *λ*. Therefore, for $𝔾(0)=R_{0}>1$, the endemic equilibrium is locally stable. Otherwise, there exists $\lambda^{\prime }$ such that $𝔾(\lambda^{\prime })=1$ has at least one root $\lambda^{\prime }=a^{\prime }\;+\;ib^{\prime }$, with $a^{\prime }\geq 0$. But $1<R_{0}=𝔾(0)<|𝔾(\lambda^{\prime })|$ which is contradiction. Therefore, for *R*_0_>1, all roots of 𝔾(λ)=1 have negative real parts, hence the endemic equilibrium is locally asymptotically stable.

### 4.3 Global stability of equilibria

**Theorem 4.2** *If R*_0_<1, *then the disease free equilibrium of the model is globally asymptotically stable.*

*Proof*: (The proof is on Appendix 2: 7) □

**Theorem 4.3** *For any* $y\in {𝒴}_{+}$, *system* (1) *has a unique solution on R*_ + _, *which depends continuously on the initial value and time. In addition,*


$$(S_{h}(t),\;V(a,t),\;I_{h}(t),\;R(b,t),\;L(t),\;S_{m}(t),\;E_{m}(t),\;I_{m}(t))\in {𝒴}_{+}\;\;for\;t\in R_{+}.$$

Actually every solution is bounded. Let $N_{h}(t)=S_{h}(t)\;+\;\int_{0}^{\infty }\xi (a)V(a,t)da\;+\;I_{h}(t)\;+\;\int_{0}^{\infty }R(b,t)db$. Then we get $\frac{dN_{h}(t)}{dt}\leq \wedge_{h}\;-\;\mu_{h}N_{h}(t)$ and hence $limsup_{t\to \infty }N_{h}(t)\leq \frac{\wedge_{h}}{\mu_{h}}$. On the other hand, if $N_{m}(t)=L(t)\;+\;S_{m}(t)\;+\;E_{m}(t)\;+\;I_{m}(t)$, then $\frac{dN_{m}(t)}{dt}\leq K$, this implies that $lim_{t\to \infty }N_{m}(t)=K$. In proposition 2.1 above, we have shown that Π is an attracting set for S. Moreover, it is also shown that Π is a positively invariant set for S. Now, by theorem 4.3, there exists a continuous semi-flow of (1), which is denoted by $\Phi :R_{+}\;\times \;{𝒴}_{+}\to {𝒴}_{+}$, with $\Phi (t,y)=(S_{h}(t),\;V(a,t),\;I_{h}(t),\;R(b,t),\;L(t),\;S_{m}(t),\;E_{m}(t),\;I_{m}(t))$ being the solution of (1) with $(S_{h0},\;V_{0}(a),\;I_{h0},\;R_{0}(b),\;L_{0},\;S_{m0},\;E_{m0},\;I_{m0})=y$.

Notation:Φ which is the semi-flow can be written as $\{\Phi (t){\}}_{t\in R_{+}}$. Now define $\delta :{𝒴}_{+}\to R_{+}$ by $\delta (S_{h},\;V,\;I_{h},\;R,\;L,\;S_{m},\;E_{m},\;I_{m})=S_{h}\left(\psi_{h}+\frac{\lambda_{mh}bI_{m}}{N_{h}}\right),\;for\;S_{h}\left(\psi_{h}+\frac{\lambda_{mh}bI_{m}}{N_{h}}\right)\in {𝒴}_{+}$.

Let ${𝒴}_{+}^{0}=\{y\in {𝒴}_{+}:\exists t_{0}\in R_{+},\;such\;that\;\delta (\Phi (t_{0},y))>0\}$. Clearly, if $y\in {𝒴}_{+}/{𝒴}_{+}^{0}$, then Φ(t,x)→DFEast→∞. By theorem 4.2 of [31], we can state and proof the following results with a little modifications.

**Theorem 4.4** *Suppose R*_0_>1. *Then the following results are true.*

*(a)* *there exists a global attractor* ℬ *for the solution semi-flow* Φ *of* (1) *in* ${𝒴}_{+}^{0}$,
*(b)* *system* (1 *is uniformly strongly* δ−persistent, *that means, there exists an* $\epsilon_{0}>0$ *such that* $liminf_{t\to \infty }\delta (\Phi (t,y))>\epsilon_{0},\;\forall y\in {𝒴}_{+}^{0}$.

ℬ can only contains points with total trajectories through them, since it must be invariant. A total trajectory of Φ is a function $Y:R\to {𝒴}_{+}$ such that $\Phi (s,Y(t))=Y(t+s),\forall t\in R\;and\;s\in R_{+}$. For a total trajectory, $V(a,t)=V(t-a)\sigma_{1},\;R(b,t)=R(t-b)\sigma_{2},\;\forall t\in R$ and $a,\;b\in R_{+}$. For a total trajectory *Y*(*t*) passing through *Y*(0) = *Y*_0_, the alpha limit is;


$$\alpha (Y_{0})=\cap_{t\leq 0}{\overset{―}{\cup Y(s)}}_{s\leq t}\subseteq ℬ\cap {𝒴}_{+}^{0}$$

**Theorem 4.5** *Suppose R*_0_>1. *Then there exists an* ϵ>0 *such that*


$$S_{h}(t),\;V(0,t),\;I_{h}(t),\;R(0,t),\;L(t),\;S_{m}(t),\;E_{m}(t),\;I_{m}(t)\geq 0,\;for\;all\;t\in R,\;where\;\left(S_{h}(t),\;V(\cdot ,t),\;I_{h}(t),\;R(\cdot ,\;t),\;L(t),\;S_{m}(t),\;E_{m}(t),\;I_{m}(t)\right)\;is\;any\;total\;trajectory\;ℬ.$$

*Proof*: To proof this theorem, we use the methods used in corollary 1 of [31] with reasonable modifications. Since Π is an attracting and invariant, there exists $M\in R_{+}$ such that for t≥M,

$S_{h}(t),\;\int_{0}^{\infty }\xi (a)V(a,\;t)da,\;I_{h}(t),\;\int_{0}^{\infty }\gamma (b)R(b,\;t)db\leq \frac{2\wedge_{h}}{\mu_{h}}$ and $L(t),\;S_{m}(t),\;E_{m}(t),\;I_{m}(t)\leq K$. For t≥M, from *S*_*h*_ equation of (1) we get $\frac{dS_{h}}{dt}\geq \wedge_{h}\;-\;\left(\mu_{h}\;+\;\frac{2\wedge_{h}\psi_{h}}{\mu_{h}}\;+\;\frac{2K\lambda_{mh}b}{N_{h}}\right)S_{h}(t)$. Hence,


$$liminf_{t\to \infty }S_{h}(t)\geq \frac{\wedge_{h}}{\mu_{h}+\frac{2\wedge_{h}\psi_{h}}{\mu_{h}}+\frac{2K\lambda_{mh}b}{N_{h}}}≊\epsilon_{1}.\;This\;implies\;S_{h}(t)\geq \epsilon_{1},\;\forall t\in R.$$

Similarly from the equation of *S*_*m*_(*t*) we have $\frac{dS_{m}}{dt}\geq S_{m0}\;-\;\left(\omega +2K+\frac{2K\lambda_{hm}b}{N_{h}}\right)$. This implies;


$$S_{m}(t)\geq \frac{S_{m0}}{\omega +2K+\frac{2K\lambda_{hm}b}{N_{h}}}≊\epsilon_{2},\;\forall t\in R.$$

$$In\;the\;same\;way\;there\;esists\;\epsilon_{3}>0,\;such\;that\;I_{h}(t)\geq \frac{\wedge_{h}}{\mu_{h}+\frac{2\wedge_{h}\beta_{1}}{\mu_{h}}+\frac{2\wedge_{h}\nu }{\mu_{h}}}≊\epsilon_{3}.$$

In a similar technique, there exists $\epsilon_{4},\;\epsilon_{5},\;and\;\epsilon_{6}$ such that;


$$L(t)\geq \frac{S_{m0}}{\omega_{l}+2K\sigma_{m}}≊\epsilon_{4},\;E_{m}(t)\geq \frac{S_{m0}}{\omega +2K\beta_{2}}≊\epsilon_{5},\;and\;I_{m}(t)\geq \frac{S_{m0}}{\omega }≊\epsilon_{6}.$$

Now, $V(0,t)=\psi_{h}S_{h}(t)$ implies there exists $\epsilon_{7}$, with $V(0,t)=\psi_{h}{§}_{h}(t)\geq \psi_{h}\epsilon_{1}=\epsilon_{7}$ and $R(0,\;t)=\beta_{1}I_{h}$ implies there exists $\epsilon_{8}>0$, such that $R(0,\;t)=\beta_{1}I_{h}\geq \beta_{1}\epsilon_{3}=\epsilon_{8},\;\forall \;t\in R$. Letting


$$\epsilon_{0}=min\{\epsilon_{1},\;\epsilon_{2},\;\epsilon_{3},\;\cdots ,\;\epsilon_{8}\},\;S_{h}(t),\;V(0,\;t),\;I_{h}(t),\;R(0,\;t),\;L(t),\;S_{m}(t),\;E_{m}(t),\;\;I_{m}(t)\geq \epsilon_{0},\;\forall \;t\in R.$$

□

**Theorem 4.6** *The endemic equilibrium of* (1) *is globally asymptotically stable in* ${𝒴}_{+}^{0}$ *for R*_0_>1.

**Proof**: (The proof is on Appendix 3: 7) □

## 5 Optimal control problem

Optimal control problems in malaria dynamics deliver an efficient outline for designing and assessing intervention strategies. By including preventive mechanisms like Long-Lasting Insecticidal Nets (LLNTs), larval source management, Indoor Residual Spraying(IRS), filling in breeding sites, larvicides and adulticides, as well as treatment of infectious humans, these models can update public health policies and resource allocation to efficiently fight malaria. In this section we present three control mechanisms, *c*_1_(*t*) that represents preventing mechanisms such as LLNTs, Surveillance, IRS, and filling in breeding sites, *c*_2_(*t*) which includes larvacide and adulticide and *c*_3_(*t*) be treatment of infectious humans. Hence, the model becomes

$$\left\{ \begin{array}{c} \frac{dS_{h}}{dt}=\Lambda_{h}-\psi_{h}S_{h}-\mu_{h}S_{h}-\frac{\lambda_{mh}b(1-c_{1}(t))I_{m}S_{h}}{N_{h}}+\int_{0}^{\infty }\xi (a)V(t,a)\;da,\\ V_{t}+V_{a}=-\xi (a)V(t,a)-\mu_{h}V(t,a),\\ V(t,0)=\psi_{h}S_{h},\\ \frac{dI_{h}}{dt}=\frac{\lambda_{mh}b(1-c_{1}(t))I_{m}S_{h}}{N_{h}}-\beta_{1}I_{h}-\mu_{h}I_{h}-(\nu +(c_{3}(t)))I_{h}+\int_{0}^{\infty }r(b)R(t,b)\;db,\\ R_{t}+R_{b}=-\mu_{h}R(t,b)-r(b)R(t,b),\\ R(t,0)=(\beta_{1}+c_{3}(t))I_{h},\\ \frac{dL}{dt}=(1-c_{2}(t))\phi \left(1-\frac{L}{c_{1}(t)K}\right)(S_{m}+E_{m}+I_{m})-\omega_{l}L-(\sigma_{m}+c_{2}(t))L,\\ \frac{dS_{m}}{dt}=\sigma_{m}L-(\omega +c_{2}(t))S_{m}-\frac{\lambda_{hm}b(1-c_{3}(t))I_{h}(1-c_{1}(t)S_{m}}{N_{h}},\\ \frac{dE_{m}}{dt}=\frac{\lambda_{hm}b(1-c_{3}(t))I_{h}(1-c_{1}(t))S_{m}}{N_{h}}-\omega E_{m}-(\beta_{2}+c_{2}(t))E_{m},\\ \frac{dI_{m}}{dt}=\beta_{2}E_{m}-(\omega +c_{2}(t))I_{m}. \end{array}\right.$$ (14)

The control variables $c_{i}\in \cup :=\{c_{i}\in L^{\infty }(0,\;T^{\prime })|\;0<c\;-\;i<l,\;l<\infty \},\;i=1,\;2,\;3$ with $L^{\infty }(0,\;T^{\prime })$ is the space of bounded almost everywhere integrable function on the set $\left[0,\;T^{\prime }\right]$ in the Lebesgue sense and ∪ is the control set. The optimal control problem intended to achieve minimizing the objective functional is expressed as;

$$J(c_{1},\;c_{2},\;c_{3})=\int_{0}^{T^{\prime }}\left[I_{h}+L+S_{m}+I_{m}+\frac{1}{2}\left(D_{1}c_{1}^{2}+D_{2}c_{2}^{2}+D_{3}c_{3}^{2}\right)\right]dt$$ (15)

where, $D_{1},\;D_{2},\;D_{3}$ are constants with positive values which balances the comparative significance for the terms in *J*. By assuming all the $c_{i}^{\prime }s$ are constants, set $c_{i}(t)=q_{i}(i=1,\;2,\;3)$ for all *t*, where *q*_*i*_ has a constant value. Hence, the controlled basic reproduction rate $R_{0}^{c}$, is given by


$$R_{0}^{c}=\frac{(1-q_{1}{)}^{2}\lambda_{hm}\lambda_{mh}\beta_{2}b^{2}\wedge_{h}S_{m}^{*}}{N_{h}^{2}\left[(q_{2}+\omega {)}^{2}(q_{2}+\omega )\beta_{2}\right]\left[\mu_{h}+q_{3}+\psi_{h}(1-\eta_{1})\right]\left[\mu_{h}+q_{3}+\nu +\beta_{1}(1-\eta_{2})\right]}.$$

Since $\frac{\partial R_{0}^{c}}{\partial q_{1}},\;\frac{\partial R_{0}^{c}}{\partial q_{2}},\;\frac{\partial R_{0}^{c}}{\partial q_{3}}<0,\;R_{0}^{c}$ is a monotonic decreasing function of $q_{1},q_{2},q_{3}$. That is increasing the function of $c_{1},\;c_{2}$ and *c*_3_ results a decreasing in $R_{0}^{c}$.

**Theorem 5.1** *There exists an optimal control variable* $c_{1}^{*},\;c_{2}^{*},\;c_{3}^{*}\in \cup$ *such that* $J(c_{1}^{*},\;c_{2}^{*},\;c_{3}^{*})=min_{c\in \cup }J(c_{1},\;c_{2},\;c_{3})$ *subject to the control system* (14) *along with the subsidiary initial and boundary conditions.*

*Proof*: Obviously the solutions are bounded and the control parameter set ∪ is closed and convex. In addition, the controlled system is bounded that implies the compactness, which we need it for the existence of the optimal control. We can also observe that the integrand in the objective functional is convex on the set ∪. Furthermore, there exists a constant $d_{1},\;d_{2},\;d_{3},\;d_{1}^{\prime },\;d_{2}^{\prime },\;d_{3}^{\prime },\;d_{1}^{\prime \prime },\;d_{2}^{\prime \prime },\;d_{3}^{\prime \prime }>1$ and positive real numbers $f_{1},\;f_{1}{\;}^{\prime },\;f1^{\prime \prime }$ and $f_{2},\;f_{2}{\;}^{\prime },\;f2^{\prime \prime }$ such that


$$J(c_{1},\;0,\;0)\geq f_{2}+f_{1}c_{1}^{d_{1}},\;J(0,\;c_{2},\;0)\geq f_{2}{\;}^{\prime }+f_{1}{\;}^{\prime }c_{2}^{d_{1}^{\prime }},\;J(0,\;0,\;c_{3})\geq f_{2}{\;}^{\prime \prime }+f_{1}{\;}^{\prime \prime }c_{3}^{d_{1}^{\prime \prime }}$$

and $J(c_{1},\;c_{2},\;c_{3})\geq f_{2}\;+\;f_{1}^{\prime }\;+\;f_{1}^{\prime }c_{2}^{d_{1}^{\prime }}\;+\;f_{1}^{\prime \prime }c_{3}^{d_{1}^{\prime \prime }}$. Therefore, our objective functional and control set satisfies all the hypotheses of theorem 13.8.1 of [32] which are necessary for the existence of an optimal control, hence, there exists an optimal control problem and this completes the proof. □

To obtain the system of optimal control, we can use the rule of Gateaux derivative from [33] to get the derivatives relative to $c_{1},\;c_{2}$ and *c*_3_. For the stated control variables, we consider another controls $c_{1}^{\varepsilon }(t)=c_{1}(t)+\varepsilon l_{1}(t),\;c_{2}^{\varepsilon }(t)=c_{2}(t)+\varepsilon l_{2}(t),\;and\;c_{3}^{\varepsilon }(t)=c_{3}(t)+\varepsilon l_{3}(t)$, where $l_{i}(i=1,\;2,\;3)$ are variation functions and ε∈(0,1). Suppose $S_{h}=S_{h}(c_{i}),\;V=V(c_{i}),\;I_{h}=I_{h}(c_{i}),\;R=R(c_{i}),\;L=L(c_{i}),\;S_{m}=S_{m}(c_{i}),\;E_{m}=E_{m}(c_{i}),\;and\;I_{m}=I_{m}(c_{i})$ and $S_{h}^{\varepsilon }=S_{h}(c_{i}^{\varepsilon }),\;V^{\varepsilon }=V(c_{i}^{\varepsilon }),\;I_{h}^{\varepsilon }=I_{h}(c_{i}^{\varepsilon }),\;R^{\varepsilon }=R(c_{i}^{\varepsilon }),\;L^{\varepsilon }=L(c_{i}^{\varepsilon }),\;S_{m}^{\varepsilon }=S_{m}(c_{i}^{\varepsilon }),\;E_{m}^{\varepsilon }=E_{m}(c_{i}^{\varepsilon }),\;and\;I_{m}^{\varepsilon }=I_{m}(c_{i}^{\varepsilon })$, then with respect to the control $c_{i}^{\varepsilon }(i=1,\;2,\;3)$, the equations of states are given by;

$$\left\{ \begin{array}{c} \frac{dS_{h}^{\varepsilon }}{dt}=\Lambda_{h}-\psi_{h}S_{h}-\mu_{h}S_{h}^{\varepsilon }-\frac{\lambda_{mh}b(1-c_{1}^{\varepsilon }(t))I_{m}^{\varepsilon }S_{h}^{\varepsilon }}{N_{h}}+\int_{0}^{\infty }\xi (a)V^{\varepsilon }(t,\;a)\;da,\\ V_{t}^{\varepsilon }+V_{a}^{\varepsilon }=-\xi (a)V^{\varepsilon }(t,\;a)-\mu_{h}V^{\varepsilon }(t,\;a),\\ V^{\varepsilon }(t,\;0)=\psi_{h}S_{h}^{\varepsilon },\\ \frac{dI_{h}^{\varepsilon }}{dt}=\frac{\lambda_{mh}b(1-c_{1}^{\varepsilon }(t))I_{m}^{\varepsilon }S_{h}^{\varepsilon }}{N_{h}}-\beta_{1}I_{h}^{\varepsilon }-\mu_{h}I_{h}^{\varepsilon }-(\nu +(c_{3}(t)))I_{h}^{\varepsilon }+\int_{0}^{\infty }r(b)R^{\varepsilon }(t,\;b)\;db,\\ R_{t}^{\varepsilon }+R_{b}^{\varepsilon }=-\mu_{h}R^{\varepsilon }(t,\;b)-r(b)R^{\varepsilon }(t,\;b),\\ R^{\varepsilon }(t,\;0)=(\beta_{1}+c_{3}^{\varepsilon }(t))I_{h}^{\varepsilon },\\ \frac{dL^{\varepsilon }}{dt}=(1-c_{2}^{\varepsilon }(t))\phi \left(1-\frac{L^{\varepsilon }}{c_{1}(t)K}\right)(S_{m}^{\varepsilon }+E_{m}^{\varepsilon }+I_{m}^{\varepsilon })-\omega_{l}L^{\varepsilon }-(\sigma_{m}+c_{2}^{\varepsilon }(t))L^{\varepsilon },\\ \frac{dS_{m}^{\varepsilon }}{dt}=\sigma_{m}L^{\varepsilon }-(\omega +c_{2}^{\varepsilon }(t))S_{m}^{\varepsilon }-\frac{\lambda_{hm}b(1-c_{3}^{\varepsilon }(t))I_{h}^{\varepsilon }(1-c_{1}^{\varepsilon }(t)S_{m}^{\varepsilon }}{N_{h}},\\ \frac{dE_{m}^{\varepsilon }}{dt}=\frac{\lambda_{hm}b(1-c_{3}^{\varepsilon }(t))I_{h}^{\varepsilon }(1-c_{1}^{\varepsilon }(t))S_{m}}{N_{h}}-\omega E_{m}^{\varepsilon }-(\beta_{2}+c_{2}^{\varepsilon }(t))E_{m}^{\varepsilon },\\ \frac{dI_{m}^{\varepsilon }}{dt}=\beta_{2}E_{m}^{\varepsilon }-(\omega +c_{2}^{\varepsilon }(t))I_{m}^{\varepsilon }, \end{array}\right.$$ (16)

where, $V^{\varepsilon }(t)=\int_{0}^{\infty }V^{\varepsilon }(a,\;t)da\;and\;R^{\varepsilon }(t)=\int_{0}^{\infty }R^{\varepsilon }(b,\;t)db$. As ε→∞, let us do the following difference quotients.

$\frac{S_{h}^{\varepsilon }(t)-S_{h}(t)}{\varepsilon }\to \overset{―}{S_{h}}(t),\frac{V^{\varepsilon }(a,\;t)-V(a,\;t)}{\varepsilon }\to \overset{―}{V}(a,\;t),\;\frac{I_{h}^{\varepsilon }(t)-I_{h}(t)}{\varepsilon }\to \overset{―}{I_{h}}(t),$ $\frac{R^{\varepsilon }(b,\;t)-R(b,\;t)}{\varepsilon }\to \overset{―}{R}(b,\;t),\;\frac{L^{\varepsilon }(t)-L(t)}{\varepsilon }\to \overset{―}{L}(t),\;\frac{S_{m}^{\varepsilon }(t)-S_{m}(t)}{\varepsilon }\to \overset{―}{S_{m}}(t),\;\frac{E_{m}^{\varepsilon }(t)-E_{m}(t)}{\varepsilon }\to \overset{―}{E_{m}}(t),$ $\frac{I_{m}^{\varepsilon }(t)-I_{m}(t)}{\varepsilon }\to \overset{―}{I_{m}}(t)$, where

$\left(\overset{―}{S_{h}}(t),\;\overset{―}{I_{h}}(t),\;\overset{―}{R}(b,\;t),\;\overset{―}{L}(t),\;\overset{―}{S_{m}}(t),\;\overset{―}{E_{m}}(t),\;\overset{―}{I_{m}}(t)\right)$ satisfies the system;

$$\left\{ \begin{array}{c} \frac{d\overset{―}{S_{h}}}{dt}=-\left(\psi_{h}+\mu_{h}+\frac{\lambda_{mh}b}{N_{h}}(1-c_{1}(t)I_{m}(t))\right)\overset{―}{S_{h}}(t)-\frac{\lambda_{mh}b}{N_{h}}(1-c_{1}(t))S_{h}(t)\overset{―}{I_{m}}(t)\\ -\frac{\lambda_{mh}b}{N_{h}}(l_{1}(t)I_{m}(t)S_{h}(t))+\int_{0}^{\infty }\xi (a)\overset{―}{V(a,t)}da,\\ {\overset{―}{V}}_{a}+{\overset{―}{V}}_{t}=-\xi (a)\overset{―}{V}(a,\;t)-\mu_{h}\overset{―}{V}(a,\;t),\;\overset{―}{V}(0,\;t)=\psi_{h}\overset{―}{S_{h}},\\ \frac{d\bar{I_{h}}}{dt}=\frac{\lambda_{mh}b(1-c_{1}(t))\overset{―}{I_{m}}S_{h}}{N_{h}}+\frac{\lambda_{mh}b(1-c_{1}(t))\overset{―}{S_{h}}I_{m}}{N_{h}}-\frac{\lambda_{mh}b}{N_{h}}l_{1}(t)I_{m}S_{h}\\ -\left(\beta_{1}+\mu_{h}+\nu +c_{3}(t)\right)\overset{―}{I_{h}}(t)-l_{3}(t)I_{h}+\int_{0}^{\infty }\gamma (b)\overset{―}{R}(b,\;t)db,\\ {\overset{―}{R}}_{a}+{\overset{―}{R}}_{t}=-\mu_{h}\overset{―}{R}(b,\;t)-\gamma (b)\overset{―}{R}(b,\;t),\;\overset{―}{R}(0,\;t)=(\beta_{1}+c_{3}(t))\overset{―}{I_{h}},\\ \frac{d\overset{―}{L}}{dt}=(1-c_{2}(t))\phi \left(1+\frac{L}{c_{1}(t)K}\right)\left(\overset{―}{S_{m}}+\overset{―}{E_{m}}+\overset{―}{I_{m}}\right)+\\ \phi \left[c_{1}(t)l_{2}(t)+c_{2}(t)l_{1}(t)-l_{1}(t)L-l_{2}(t)-\left(\frac{1-c_{2}(t)}{c_{1}(t)K}\right)L\right](S_{m}+E_{m}+I_{m})\\ -(\omega_{l}+\sigma_{m}+c_{2}(t))\overset{―}{L}-l_{2}(t)L,\\ \frac{d\overset{―}{S_{m}}}{dt}=\sigma_{m}L-(\omega +c_{2}(t))\bar{S_{m}}-\frac{\lambda_{mh}b}{N_{h}}(1-c_{1}(t))(1-c_{3}(t))\left(\overset{―}{S_{m}}I_{h}+\overset{―}{I_{h}}S_{m}\right)\\ \frac{\lambda_{hm}b}{N_{h}}\left[(1-c_{1}(t))l_{3}(t)+(1-c_{3}(t)l_{1}(t))\right]S_{m}I_{h}-l_{2}(t)S_{m},\\ \frac{d\;\overset{―}{E_{m}}}{dt}=\frac{\lambda_{mh}b}{N_{h}}(1-c_{1}(t))(1-c_{3}(t))\left(\overset{―}{S_{m}}I_{h}+\overset{―}{I_{h}}S_{m}\right)-(\omega +\beta_{2}+c_{2}(t))\overset{―}{E_{m}}\\ -\frac{\lambda_{hm}b}{N_{h}}\left[(1-c_{1}(t))l_{3}(t)+(1-c_{3}(t)l_{1}(t))\right]S_{m}I_{h}-l_{2}(t)E_{m},\\ \frac{d\overset{―}{I_{m}}}{dt}=\beta_{2}\overset{―}{E_{m}}-(\omega +c_{2}(t))\overset{―}{I_{m}}-l_{2}(t)I_{m}. \end{array}\right.$$ (17)

To derive the adjoint equations, we use the Pontryagin Maximum Principle (PMP), particularily the condition $\langle 0,\;\lambda_{i}\rangle$ (since $\delta_{x}(T)=0$ for fixed final time problems). Therefore, to get the respective adjoint equations, we put the above equations in the form,


$$\langle \frac{d\overset{―}{S_{h}}}{dt}+\left(\psi_{h}+\mu_{h}+\frac{\lambda_{mh}b}{N_{h}}(1-c_{1}(t)I_{m}(t))\right)\overset{―}{S_{h}}(t)+\frac{\lambda_{mh}b}{N_{h}}(1-c_{1}(t))S_{h}(t)\overset{―}{I_{m}}(t)\;+\frac{\lambda_{mh}b}{N_{h}}(l_{1}(t)I_{m}(t)S_{h}(t))-\int_{0}^{\infty }\xi (a)\overset{―}{V}(a,\;t)da,\;\lambda_{1}(t)\rangle =0$$

This gives


$$\langle \overset{―}{S_{h}}(t),-\frac{d\lambda_{1}}{dt}+\left[\left(\psi_{h}+\mu_{h}+\frac{\lambda_{mh}b}{N_{h}}(1-c_{1}(t))\right)I_{m}\right]\lambda_{1}(t)\rangle +\int_{0}^{T}\lambda_{1}(t)\frac{\lambda_{mh}b}{N_{h}}(1-c_{1}(t))S_{h}\overset{―}{I_{m}}dt\;\;+\int_{0}^{T}\frac{\lambda_{mh}b}{N_{h}}l_{1}(t)I_{m}S_{h}\lambda_{1}(t)dt-\int_{0}^{T}\int_{0}^{\infty }\xi (a)\overset{―}{V}(a,\;t)\lambda_{1}(t)dadt=0,\;with\;\lambda_{1}(T)=0,\overset{―}{S_{h}}(0)=0,\;and\;\langle h,g\rangle =\int_{0}^{T}hgdt,\;\langle \langle {\bar{V}}_{a}+{\overset{―}{V}}_{t}+\xi (a)\overset{―}{V}(a,\;t)+\mu_{h}\overset{―}{V}(a,\;t),\;\lambda_{2}(a,\;t)\rangle \rangle =0.\;This\;gives\;\langle \langle \overset{―}{V}(a,\;t),-\frac{\partial \lambda_{2}(a,\;t)}{\partial a}-\frac{\partial \lambda_{2}(a,\;t)}{\partial }+(\mu_{h}+\xi (a))\lambda_{2}(a,\;t)\rangle \rangle -\int_{0}^{T}\psi_{h}\lambda_{2}(0,\;t)\overset{―}{S_{h}}(t)dt=0,$$

$$with\;\overset{―}{V}(a,\;0)=\overset{―}{V}(t,\;\infty )=0,\;\lambda_{2}(a,\;T)=0\;and\;\langle \langle h,\;g\rangle \rangle =\int_{0}^{T}\int_{0}^{\infty }hgdadt.\;\;\;\;\;\;\;\;\;\;\;\langle \frac{d\overset{―}{I_{h}}}{dt}-\frac{\lambda_{mh}b(1-c_{1}(t))\overset{―}{I_{m}}S_{h}}{N_{h}}-\frac{\lambda_{mh}b(1-c_{1}(t))\overset{―}{S_{h}}I_{m}}{N_{h}}+\frac{\lambda_{mh}b}{N_{h}}l_{1}(t)I_{m}S_{h}+\left(\beta_{1}+\mu_{h}+\nu +c_{3}(t)\right)\;\;\overset{―}{I_{h}}(t)+l_{3}(t)I_{h}-\int_{0}^{\infty }\gamma (b)\overset{―}{R}(b,\;t)db,\lambda_{3}(t)\rangle =0.$$

This can be written as;


$$\langle \overset{―}{I_{h}}(t),-\frac{d\lambda_{3}(t)}{dt}+(\beta_{1}+\mu_{h}+\nu +c_{3}(t)\lambda_{3}(t))\rangle -\int_{0}^{T}\frac{\lambda_{mh}b}{N_{h}}(1-c_{1}(t))\overset{―}{I_{m}}(t)S_{h}\lambda_{3}(t)dt\;\;-\int_{0}^{T}\frac{\lambda_{mh}b}{N_{h}}(1-c_{1}(t))\overset{―}{S_{h}}(t)I_{m}\lambda_{3}(t)dt+\int_{0}^{T}\frac{\lambda_{mh}b}{N_{h}}l_{1}(t)I_{m}S_{h}\lambda_{3}(t)dt+\int_{0}^{T}l_{3}(t)I_{h}\lambda_{3}(t)dt\;\;-\int_{0}^{T}\int_{0}^{\infty }\gamma (b)\bar{R}(b,\;t)\lambda_{3}(t)dbdt=0,\;with\;\lambda_{3}(T)=\bar{I_{h}}(0)=0.\;\langle \langle {\overset{―}{R}}_{a}+{\bar{R}}_{t}+\mu_{h}\bar{R}(b,\;t)+\gamma (b)\bar{R}(b,\;t),\lambda_{4}(b,t)\rangle \rangle =0.$$

This implies


$$\langle \langle \overset{―}{R}(b,\;t),-\frac{\partial \lambda_{4}(b,\;t)}{\partial b}-\frac{\partial \lambda_{4}(b,t)}{\partial t}+(\mu_{h}+\gamma (b))\lambda_{4}(b,\;t)\rangle \rangle -\int_{0}^{T}(\beta_{1}+c_{3}(t))\lambda_{4}(0,\;t)\overset{―}{I_{h}}(t)dt=0,\;with\;\overset{―}{R}(b,\;0)=\overset{―}{R}(t,\infty )=\lambda_{4}(T,\;b)=0.$$

$$\langle \frac{d\overset{―}{L}}{dt}-(1-c_{2}(t))\phi \left(1+\frac{L}{c_{1}(t)K}\right)\left(\overset{―}{S_{m}}+\overset{―}{E_{m}}+\overset{―}{I_{m}}\right)\;\;-\phi \left[c_{1}(t)l_{2}(t)+c_{2}(t)l_{1}(t)-l_{1}(t)L-l_{2}(t)-\left(\frac{1-c_{2}(t)}{c_{1}(t)K}\right)L\right]\;(S_{m}+E_{m}+I_{m})+(\omega_{l}+\sigma_{m}+c_{2}(t))\overset{―}{L}+l_{2}(t)L,\;\lambda_{5}(t)\rangle =0.\;⟹\langle \overset{―}{L}(t),\;-\frac{d\lambda_{5}(t)}{dt}+(\omega_{l}+\sigma_{m}+c_{2}(t))\lambda_{5}(t)\rangle +\int_{0}^{T}l_{2}(t)L\lambda_{5}(t)dt\;\;-\int_{0}^{T}(1-c_{2}(t))\phi \left(1+\frac{L}{c_{1}(t)K}\right)\left(\overset{―}{S_{m}}(t)+\overset{―}{E_{m}}(t)+\overset{―}{I_{m}}(t)\right)\lambda_{5}(t)dt\;\;-\int_{0}^{T}\phi \lambda_{5}(t)\left[c_{1}(t)l_{2}(t)+c_{2}(t)l_{1}(t)-l_{1}(t)L-l_{2}(t)-\left(\frac{1-c_{2}(t)}{c_{1}(t)K}\right)L\right]\left(S_{m}+E_{m}+I_{m}\right)dt=0,\;with\;\lambda_{5}(t)=\overset{―}{L}(0)=0.\;\langle \frac{d\overset{―}{S_{m}}}{dt}-\sigma_{m}L+(\omega +c_{2}(t))\overset{―}{S_{m}}+\frac{\lambda_{mh}b}{N_{h}}(1-c_{1}(t))(1-c_{3}(t))\left(\overset{―}{S_{m}}I_{h}+\overset{―}{I_{h}}S_{m}\right)\;\;-\frac{\lambda_{hm}b}{N_{h}}\left[(1-c_{1}(t))l_{3}(t)+(1-c_{3}(t)l_{1}(t))\right]S_{m}I_{h}+l_{2}(t)S_{m},\lambda_{6}(t)\rangle =0.\;Hence,\;\langle \overset{―}{S_{m}}-\frac{d\lambda_{6}(t)}{dt}+(\omega +c_{2}(t))\lambda_{6}(t)\rangle -\int_{0}^{T}\sigma_{m}\bar{L}\lambda_{6}(t)dt+\int_{0}^{T}\frac{\lambda_{mh}b}{N_{h}}(1-c_{1}(t))(1-c_{3}(t))$$

$$\left(\overset{―}{S_{m}}(t)I_{h}+\overset{―}{I_{h}}(t)S_{m}\right)\lambda_{6}(t)dt-\int_{0}^{T}\frac{\lambda_{mh}b}{N_{h}}\lambda_{6}(t)\left[(1-c_{1}(t))l_{3}(t)+(1-c_{3}(t))l_{1}(t)\right]S_{m}I_{h}dt\;\;+\int_{0}^{T}l_{2}(t)S_{m}\lambda_{6}(t)dt\;with\;\lambda_{6}(T)=\overset{―}{S_{m}}(0)=0.\;\langle \frac{d\overset{―}{E_{m}}}{dt}-\frac{\lambda_{mh}b}{N_{h}}(1-c_{1}(t))(1-c_{3}(t))\left(\overset{―}{S_{m}}I_{h}+\overset{―}{I_{h}}S_{m}\right)+(\omega +\beta_{2}+c_{2}(t))\overset{―}{E_{m}}\;\;+\frac{\lambda_{hm}b}{N_{h}}\left[(1-c_{1}(t))l_{3}(t)+(1-c_{3}(t)l_{1}(t))\right]S_{m}I_{h}+l_{2}(t)E_{m},\;\lambda_{7}(t)\rangle =0.$$

From this we get


$$\langle \overset{―}{E_{m}}(t),-\frac{d\lambda_{7}(t)}{dt}+(\omega +\beta_{2}+c_{2}(t))\lambda_{7}(t)\rangle -\int_{0}^{T}\frac{\lambda_{hm}b}{N_{h}}(1-c_{1}(t))(1-c_{3}(t))\;\left[\overset{―}{S_{m}}(t)I_{h}+\overset{―}{I_{h}}(t)S_{m}\right]\lambda_{7}(t)dt\;\;+\int_{0}^{T}\frac{\lambda_{hm}b}{N_{h}}\left[(1-c_{1}(t))l_{3}(t)+(1-c_{3}(t))l_{1}(t)\right]S_{m}I_{h}\lambda_{7}(t)dt+\int_{0}^{T}l_{2}(t)E_{m}\lambda_{7}(t)dt=0,\;\;with\;\lambda_{7}(T)=\overset{―}{E_{m}}(0)=0,\langle \frac{d\overset{―}{I_{m}}}{dt}-\beta_{2}\overset{―}{E_{m}}+(\omega +c_{2}(t))\overset{―}{I_{m}}+l_{2}(t)I_{m},\;\lambda_{8}(t)\rangle =0.\;\;\;This\;gives\;us;\langle \overset{―}{I_{m}}(t),\;-\frac{d\lambda_{8}(t)}{dt}+(\omega +c_{2}(t))\lambda_{8}(t)\rangle -\int_{0}^{T}\beta_{2}\overset{―}{E_{m}}(t)\lambda_{8}(t)dt\;\;+\int_{0}^{T}l_{2}(t)I_{m}(t)\lambda_{8}(t)dt=0,\;with\;\lambda_{8}(T)=\overset{―}{I_{m}}(0)=0$$

Now from (15) above, we can derive the adjoint equations by defining a Lagrangian ℒ as;


$$ℒ\left(S_{h},\;V,\;I_{h},\;R,\;L,\;S_{m},\;E_{m},\;I_{m}\right)=\int_{0}^{T}\left[\overset{―}{I_{h}}+\overset{―}{L}+\overset{―}{S_{m}}+\overset{―}{I_{m}}+\overset{―}{E_{m}}+\frac{1}{2}\left(D_{1}c_{1}^{2}+D_{2}c_{2}^{2}+D_{3}c_{3}^{2}\right)\right]dt\;=-\lambda_{1}(t)\int_{0}^{T}[\frac{d\overset{―}{S_{h}}}{dt}+\left(\psi_{h}+\mu_{h}+\frac{\lambda_{mh}b}{N_{h}}(1-c_{1}(t))I_{m}\right)\bar{S_{h}}(t)\;\;+\frac{\lambda_{mh}b}{N_{h}}(1-c_{1}(t))S_{h}\overset{―}{I_{m}}(t)+\frac{\lambda_{mh}b}{N_{h}}l_{1}(t)S_{h}I_{m}]+\lambda_{1}(t)\int_{0}^{T}\left[\int_{0}^{\infty }\xi (a)\overset{―}{V}(a,\;t)da\right]dt\;-\lambda_{2}(a,\;t)\int_{0}^{T}\int_{0}^{\infty }\left[({\overset{―}{V}}_{a}+{\overset{―}{V}}_{t})(a,\;t)+\xi (a)\overset{―}{V}(a,\;t)+\mu_{h}\overset{―}{V}(a,\;t)\right]dadt\;-\lambda_{3}(t)\int_{0}^{T}\left[\frac{d\overset{―}{I_{h}}}{dt}-\frac{\lambda_{mh}b(1-c_{1}(t))S_{h}\overset{―}{I_{m}}(t)}{N_{h}}-\frac{\lambda_{mh}b}{N_{h}}(1-c_{1}(t))I_{m}\overset{―}{S_{h}}(t)+\frac{\lambda_{mh}b}{N_{h}}l_{1}(t)I_{m}S_{h}\right]dt\;-\lambda_{3}(t)\int_{0}^{T}\left[(\beta_{1}+\mu_{h}+\nu +c_{3}(t))\overset{―}{I_{h}}(t)-l_{3}(t)I_{h}-\int_{0}^{\infty }\gamma (b)\overset{―}{R}(b,\;t)db\right]dt\;-\lambda_{4}(b,\;t)\int_{0}^{T}\int_{0}^{\infty }\left[({\overset{―}{R}}_{b}+{\overset{―}{R}}_{t})(b,\;t)+(\mu_{h}+\gamma (b))\overset{―}{R}(b,\;t)\right]\;-\lambda_{5}(t)\int_{0}^{T}[\frac{d\overset{―}{L}}{dt}-(1-c_{2}(t))\phi \left(1+\frac{L}{c_{1}(t)K}\right)(\overset{―}{S_{m}}(t)+\bar{E_{m}}(t)+\overset{―}{I_{m}}(t))\;\;-\phi \left(c_{1}(t)l_{2}(t)+c_{2}(t)l_{1}(t)-l_{1}(t)L\right)]dt\;+\lambda_{5}(t)\int_{0}^{T}\left[\phi \left[-l_{2}(t)-\left(\frac{1-c_{2}(t)}{c_{1}(t)K}\right)\overset{―}{L}(t)\right]\left(S_{m}+E_{m}+I_{m}\right)+\left(\omega_{l}+\sigma_{m}+c_{2}(t)\right)\overset{―}{L}+l_{2}(t)L\right]dt\;-\lambda_{6}(t)\int_{0}^{T}[\frac{d\overset{―}{S_{m}}(t)}{dt}-\sigma_{m}\overset{―}{L}(t)+(\omega +c_{2}(t))\overset{―}{S_{m}}(t)\;\;+\frac{\lambda_{hm}b}{N_{h}}(1-c_{1}(t))(1-c_{3}(t))\left(\overset{―}{S_{m}}(t)I_{h}+\overset{―}{I_{h}}(t)S_{m}\right)]dt\;-\lambda_{6}(t)\int_{0}^{T}\left[-\frac{\lambda_{hm}b}{N_{h}}\left((1-c_{1}(t)l_{3}(t))+(1-c_{3}(t)l_{1}(t))\right)S_{m}I_{h}-l_{2}(t)S_{m}\right]dt-\lambda_{7}(t)$$

$$\int_{0}^{T}\left[\frac{d\overset{―}{E_{m}}}{dt}-\frac{\lambda_{hm}b}{N_{h}}(1-c_{1}(t))(1-c_{3}(t))\left(\overset{―}{S_{m}}(t)I_{h}+\overset{―}{I_{h}}(t)S_{m}\right)+(\omega +\beta_{2}+c_{2}(t))\overset{―}{E_{m}}(t)\right]dt\;\;-\lambda_{7}(t)\int_{0}^{T}\left[\frac{\lambda_{hm}b}{N_{h}}\left((1-c_{1}(t)l_{3}(t))+(1-c_{3}(t))l_{1}(t)\right)S_{m}I_{h}+l_{2}(t)E_{m}\right]dt\;\;-\lambda_{8}(t)\int_{0}^{T}\left[\frac{d\overset{―}{I_{m}}(t)}{dt}-\beta_{2}\overset{―}{E_{m}}+(\omega +c_{2}(t))\overset{―}{I_{m}}(t)+l_{2}(t)I_{m}(t)\right]dt.$$

To derive the adjoint (co-state) equations, we apply Pontryagin’s Maximum Principle, which provides necessary conditions for optimality in control problems. Specifically, the adjoint system is obtained by setting the partial derivatives of the Lagrangian functional ℒ with respect to the state variables equal to zero. This leads to the condition:


$$\frac{\partial ℒ}{\partial \bar{S_{h}}}=\frac{\partial ℒ}{\partial \bar{V}}=\frac{\partial ℒ}{\partial \bar{I_{h}}}=\frac{\partial ℒ}{\partial \bar{R}}=\frac{\partial ℒ}{\partial \bar{L}}=\frac{\partial ℒ}{\partial \bar{S_{m}}}=\frac{\partial ℒ}{\partial \bar{E_{m}}}=\frac{\partial ℒ}{\partial \bar{I_{m}}}=0,$$

which yields the system of adjoint equations governing the evolution of the costate variables. Therefore, the adjoint equations are obtained as;

$$\left\{ \begin{array}{c} \frac{d\lambda_{1}}{dt}=\left(\psi_{h}+\mu_{h}+\frac{\lambda_{mh}b}{N_{h}}(1-c_{1}(t))\right)I_{m}\lambda_{1}(t)+\frac{\lambda_{mh}b}{N_{h}}(1-c_{1}(t)I_{m}(t)\lambda_{3}(t))-\psi_{h}\lambda_{2}(0,\;t),\\ \frac{\partial \lambda_{2}}{\partial a}+\frac{\partial \lambda_{2}}{\partial t}=\left(\mu_{h}+\xi (a)\right)\lambda_{2}(a,\;t)-\lambda_{1}(t)\xi (a),\\ \frac{d\lambda_{3}}{dt}=\left(\beta_{1}+\mu_{h}+\nu +c_{3}(t)\right)\lambda_{3}(t)+\frac{\lambda_{hm}b}{N_{h}}(1-c_{1}(t))(1-c_{3}(t))\lambda_{6}(t)\\ -\frac{\lambda_{mh}b}{N_{h}}(1-c_{1}(t))(1-c_{3}(t))S_{m}\lambda_{7}(t)\\ -(\beta_{1}+c_{3}(t))\lambda_{4}(0,\;t)-1,\\ \frac{\partial \lambda_{4}}{\partial b}+\frac{\partial \lambda_{4}}{\partial t}=-\lambda_{3}(t)\gamma (b)+(\mu_{h}+\gamma (b))\lambda_{4}(b,\;t),\\ \frac{d\lambda_{5}}{dt}=\left(\omega_{l}+\sigma_{m}+c_{2}(t)\right)\lambda_{5}(t)-\left(\frac{1-c_{2}(t)}{c_{1}(t)K}\right)\left(S_{m}+E_{m}+I_{m}\right)\lambda_{5}(t)-\sigma_{m}\lambda_{6}(t)-1,\\ \frac{d\lambda_{6}}{dt}=(\omega +c_{2}(t))\lambda_{6}(t)-(1-c_{2}(t))\phi \left(1+\frac{L}{c_{1}(t)K}\right)\lambda_{5}(t)\\ +\frac{\lambda_{hm}b}{N_{h}}(1-c_{1}(t))(1-c_{3}(t))I_{h}\lambda_{6}(t)-\frac{\lambda_{mh}b}{N_{h}}(1-c_{1}(t))(1-c_{3}(t))I_{h}\lambda_{7}(t)-1,\\ \frac{d\lambda_{7}}{dt}=(\omega +\beta_{2}+c_{2}(t))\lambda_{7}(t)-(1-c_{2}(t))\phi \left(1+\frac{L}{c_{1}(t)K}\right)\lambda_{5}(t)-\beta_{2}\lambda_{8}(t)-1,\\ \frac{d\lambda_{8}}{dt}=(\omega +\beta_{2})\lambda_{8}(t)+\frac{\lambda_{mh}b}{N_{h}}(1-c_{1}(t))S_{h}\lambda_{1}(t)-\frac{\lambda_{mh}b}{N_{h}}(1-c_{1}(t))S_{h}\lambda_{3}(t)\\ -(1-c_{2}(t))\phi \left(1+\frac{L}{c_{1}(t)K}\right)\lambda_{5}(t)+l_{2}(t)\lambda_{8}(t)-1. \end{array}\right.$$ (18)

**Theorem 5.2.** *If the optimal controls which minimizes* $J(c_{1},\;c_{2},\;c_{3})$ *are* $c_{1}^{*},\;c_{2}^{*},\;c_{3}^{*}$ *and*

$\left(S_{h}^{*},\;V^{*},\;I_{h}^{*},\;R^{*},\;L^{*},\;S_{m}^{*},\;E_{m}^{*},\;I_{m}^{*}\right),$

$\left(\lambda_{1}(t),\;\lambda_{2}(a,\;t),\lambda_{3}(t),\;\lambda_{4}(b,\;t),\;\lambda_{5}(t),\;\lambda_{6}(t),\lambda_{7}(t),\;\lambda_{8}(t)\right)$ *are the corresponding state variables and adjoint variables, respectively, then*

$$\left\{ \begin{array}{c} c_{1}^{*}=Min\{max\{c_{1},\;\overset{―}{c_{1}}\},\;c_{1}^{max}\}\\ c_{2}^{*}=Min\{max\{c_{2},\;\overset{―}{c_{2}}\},\;c_{2}^{max}\}\\ c_{3}^{*}=Min\{max\{c_{3},\;\overset{―}{c_{3}}\},\;c_{3}^{max}\} \end{array}\right.$$ (19)

*with* $c_{1}^{*},\;c_{2}^{*},\;c_{3}^{*}\in L^{\infty }(0,\;T)$ *and* $\bar{c_{1}},\;\overset{―}{c_{2}},\;\overset{―}{c_{3}}$ *are in Eq* (21) *below.*

*Proof*: $\frac{\partial L}{\partial c_{i}}=0,\;i=1,\;2,\;3$ optimal conditions. Then

$$\left\{ \begin{array}{c} \frac{\partial L}{\partial c_{1}}=D_{1}c_{1}(t)+\frac{\lambda_{mh}b}{N_{h}}\overset{―}{S_{m}}(t)\bar{I_{h}}(t)(\lambda_{1}(t)-\lambda_{3}(t))+\frac{\lambda_{hm}b}{N_{h}}(1-c_{3}(t))S_{m}I_{h}(\lambda_{6}(t)-\lambda_{7}(t))=0\\ \frac{\partial L}{\partial c_{2}}=D_{2}c_{2}(t)-\left(L+\phi \left(\overset{―}{S_{m}}(t)+\bar{E_{m}}(t)+\overset{―}{I_{m}}(t)\right)\right)\lambda_{5}(t)-\overset{―}{S_{m}}(t)\lambda_{6}(t)-\bar{E_{m}}(t)\lambda_{7}(t)\\ -\overset{―}{I_{m}}(t)\lambda_{8}(t)=0,\\ \frac{\partial L}{\partial c_{3}}=D_{3}c_{3}(t)-\overset{―}{I_{h}}(t)\lambda_{3}(t)+\frac{\lambda_{hm}b}{N_{h}}(1-c_{1}(t))\overset{―}{S_{m}}(t)\overset{―}{I_{h}}(t)(\lambda_{6}(t)-\lambda_{7}(t))=0. \end{array}\right.$$ (20)

Solving the above system for $c_{i}^{\prime }$s we get

$$\overset{―}{c_{1}}=\frac{\begin{array}{c} \frac{\lambda_{hm}b}{N_{h}}\frac{1}{D_{3}}I_{h}^{2}S_{m}\lambda_{3}(\lambda_{6}-\lambda_{7})-{\left(\frac{\lambda_{hm}b}{N_{h}}\right)}^{2}\frac{1}{D_{3}}S_{m}^{2}I_{h}^{2}(\lambda_{6}-\lambda_{7}{)}^{2}\\ -\frac{\lambda_{hm}b}{N_{h}}S_{m}I_{h}(\lambda_{6}-\lambda_{7})-\frac{\lambda_{mh}b}{N_{h}}S_{m}I_{h}(\lambda_{1}-\lambda_{3}) \end{array}}{D_{1}-{\left(\frac{\lambda_{hm}b}{N_{h}}\right)}^{2}\frac{1}{D_{3}}S_{m}^{2}I_{h}^{2}(\lambda_{6}-\lambda_{7}{)}^{2}}$$ (21)

$$\overset{―}{c_{2}}=\frac{L+\phi \left(\bar{S_{m}}(t)+\overset{―}{E_{m}}(t)+\overset{―}{I_{m}}(t)\right)\lambda_{5}(t)+\overset{―}{S_{m}}(t)\lambda_{6}(t)+\overset{―}{E_{m}}(t)\lambda_{7}(t)+\overset{―}{I_{m}}(t)\lambda_{8}(t)}{D_{2}}.$$ (22)

$$\overset{―}{c_{3}}=\frac{\overset{―}{I_{h}}(t)\lambda_{3}(t)-\frac{\lambda_{hm}b}{N_{h}}(1-c_{1}(t))\overset{―}{S_{m}}(t)\overset{―}{I_{h}}(t)(\lambda_{6}(t)-\lambda_{7}(t))}{D_{3}}.$$ (23)

Therefore, the values of $c_{1}^{*},\;c_{2}^{*},\;c_{3}^{*}$ are in Eq (19) above, where $c_{1}^{max},\;c_{2}^{max},\;c_{3}^{max}$ respectively are the corresponding upper bounds for the three controls. □

We can use Ekeland’s principle [33], to prove the existence problem of optimal control and to get the minimize sequences of approximate functions. Using [23], it is assumed that there exists a set of sequences that satisfy, the objective function


$$J_{\varepsilon }(c_{1},\;c_{2},\;c_{3})=J(c_{1},\;c_{2},\;c_{3})+\sqrt{\varepsilon }\left(||c_{1}^{\varepsilon }-c_{1}|{|}_{{ℒ}^{1}(0,\;T)}+||c_{2}^{\varepsilon }-c_{2}|{|}_{{ℒ}^{1}(0,\;T)}+||c_{3}^{\varepsilon }-c_{3}|{|}_{{ℒ}^{1}(0,\;T)}\right).$$

**Theorem 5.3** *Suppose* $\left(c_{1}^{\varepsilon },\;c_{2}^{\varepsilon },\;c_{3}^{\varepsilon }\right)$ *is minimizers for* $J_{\varepsilon }(c_{1},\;c_{2},\;c_{3})$, *then*

$c_{1}^{\varepsilon }=min\{max\{0,\;{\overset{―}{c_{1}}}^{\varepsilon }\},\;c_{1}^{max}\},\;c_{2}^{\varepsilon }=min\{max\{0,\;{\overset{―}{c_{2}}}^{\varepsilon }\},\;c_{2}^{max}\},\;c_{3}^{\varepsilon }=min\{max\{0,\;{\overset{―}{c_{3}}}^{\varepsilon }\},\;c_{3}^{max}\}$, *where*


$${\overset{―}{c_{1}}}^{\varepsilon }=\frac{\begin{array}{c} \frac{\lambda_{hm}b}{N_{h}}\frac{1}{D_{3}}(I_{h}^{\varepsilon }{)}^{2}S_{m}^{\varepsilon }\lambda_{3}^{\varepsilon }(\lambda_{6}^{\varepsilon }-\lambda_{7}^{\varepsilon })-{\left(\frac{\lambda_{hm}b}{N_{h}}\right)}^{2}\frac{1}{D_{3}}(S_{m}^{\varepsilon }{)}^{2}(I_{h}^{\varepsilon }{)}^{2}(\lambda_{6}^{\varepsilon }-\lambda_{7}^{\varepsilon }{)}^{2}\\ -\frac{\lambda_{hm}b}{N_{h}}S_{m}^{\varepsilon }I_{h}^{\varepsilon }(\lambda_{6}^{\varepsilon }-\lambda_{7}^{\varepsilon })-\frac{\lambda_{mh}b}{N_{h}}S_{m}^{\varepsilon }I_{h}^{\varepsilon }(\lambda_{1}^{\varepsilon }-\lambda_{3}^{\varepsilon }) \end{array}}{D_{1}-{\left(\frac{\lambda_{hm}b}{N_{h}}\right)}^{2}\frac{1}{D_{3}}(S_{m}^{\varepsilon }{)}^{2}(I_{h}^{\varepsilon }{)}^{2}(\lambda_{6}^{\varepsilon }-\lambda_{7}^{\varepsilon }{)}^{2}}-\frac{\sqrt{\varepsilon }\gamma_{1}^{\varepsilon }}{D_{1}}$$

$${\overset{―}{c_{2}}}^{\varepsilon }=\frac{L^{\varepsilon }+\phi \left(\overset{―}{S_{m}^{\varepsilon }}(t)+\bar{E_{m}^{\varepsilon }}(t)+\overset{―}{I_{m}^{\varepsilon }}(t)\right)\lambda_{5}^{\varepsilon }(t)+\overset{―}{S_{m}^{\varepsilon }}(t)\lambda_{6}^{\varepsilon }(t)+\overset{―}{E_{m}^{\varepsilon }}(t)\lambda_{7}^{\varepsilon }(t)+\overset{―}{I_{m}^{\varepsilon }}(t)\lambda_{8}^{\varepsilon }(t)}{D_{2}}-\frac{\sqrt{\varepsilon }\gamma_{2}^{\varepsilon }}{D_{2}}.$$

$${\overset{―}{c_{3}}}^{\varepsilon }=\frac{\bar{I_{h}^{\varepsilon }}(t)\lambda_{3}^{\varepsilon }(t)-\frac{\lambda_{hm}b}{N_{h}}(1-c_{1}(t))\bar{S_{m}^{\varepsilon }}(t)\bar{I_{h}^{\varepsilon }}(t)(\lambda_{6}^{\varepsilon }(t)-\lambda_{7}^{\varepsilon }(t))}{D_{3}}-\frac{\sqrt{\varepsilon }\gamma_{3}^{\varepsilon }}{D_{3}}.$$

*and* $\left(\gamma_{1}^{\varepsilon },\;\gamma_{2}^{\varepsilon },\;\gamma_{3}^{\varepsilon }\right)$ *belongs to* ${ℒ}^{\infty }(0,\;T)$ *such that* $\left|\gamma_{i}^{\varepsilon }|\leq 1(i=1,\;2,\;3\right)$ *for all* t∈(0,T).

*Proof*: The proof of this theorem is exactly similar with the proof of the previous theorem 5.2. □

**Theorem 5.4.** *If* $\frac{T}{D_{1}},\;\frac{T}{D_{2}}$ *and* $\frac{T}{D_{3}}$ *are sufficiently small, then there exists a unique optimal control* $\left(c_{1}^{*},\;c_{2}^{*},\;c_{3}^{*}\right)$ *minimizing* $J(c_{1},\;c_{2},\;c_{3})$.

*Proof*: The proof of this theorem follows from theorem 5.4 of [23]. □

## 6 Numerical findings

In the current section, we present some computational experiments to explore the effect of temperature on the transmission dynamics of malaria, to show stability of both infection-free-equilibrium and endemic steady state and to explain the influence of optimal controls considered on malaria disease. Besides the temperature dependent parameters stated in Sect 3, we take the values of the basic parameters in the model (1) from [23] as $\mu_{h}=0.000346,\;\nu =0.0035,\;\beta_{1}=0.143,\;\gamma (b)=\left(0.003+sin(b-10)\frac{3\pi }{20000}\right)$, and


$$\xi (a)=\left\{ \begin{array}{c} 0,\;\;0<a<10,\\ 0.667(a-10{)}^{2}e^{-0.6(a-10)},\;\;10\leq a<30,\\ 0.0185,\;\;a\geq 30. \end{array}\right.$$

**Fig 12:**
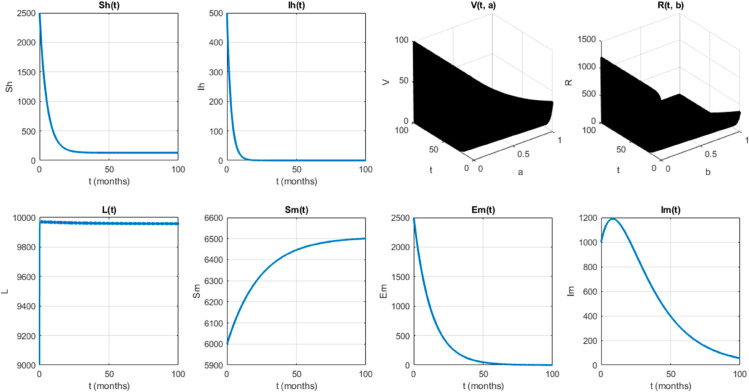
Time evolution of the human and mosquito compartments in the absence of control interventions. The susceptible and infected human populations decline rapidly as individuals transition into vaccinated and recovered states.

**Fig 13:**
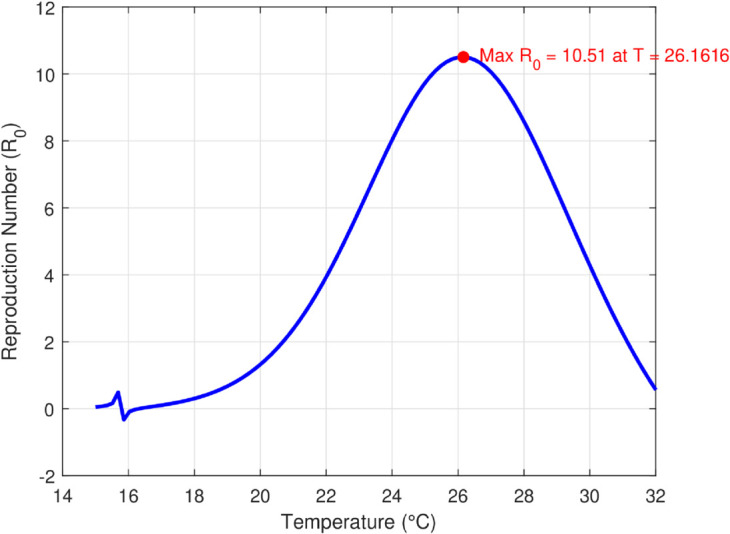
Temperature-dependent variation of the basic reproduction number, which peaks at 10.51 when *T* = 26.1616^*o*^*C.* The figure highlights the existence of an optimal temperature for malaria transmission, beyond which *R*_0_ declines, indicating reduced transmission potential at both lower and higher temperatures.

**Fig 14:**
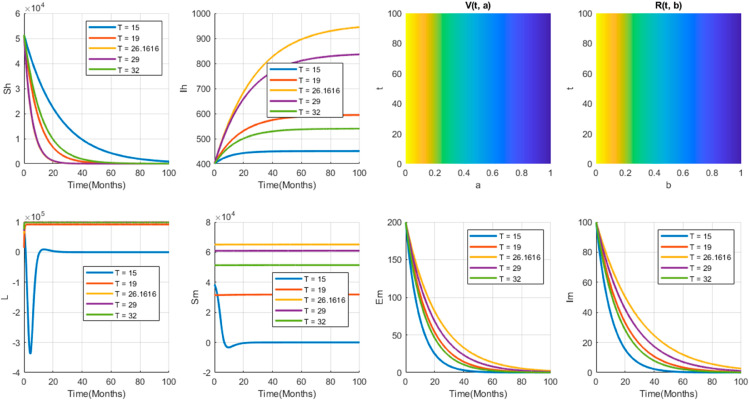
Time evolution of model compartments under varying temperature conditions. The figure shows that temperature value, *T* = 26.1616^*o*^*C* accelerate transmission dynamics, leading to higher peaks in infection prevalence, indicating the strong temperature sensitivity of malaria spread.

We take the values of other parameters from [24] as $\lambda_{mh}=0.062,\;\lambda_{hm}=0.24$ and $\beta_{2}=0.0379$. We choose $\wedge_{h}=25,\;\psi_{h}=0.0006,\;$ temperature (*T* = 26.1616^0^*C*) which is the optimum temperature for mosquitoes, and initial conditions $S_{h0}=2500,\;I_{h0}=500,\;S_{m0}=6000,\;E_{m0}=2500,\;I_{m0}=1000,\;L_{0}=9000,\;V_{0}=100e^{-a},\;R_{0}=1000e^{-b},\;K=200\;and\;N_{h}=3000$ to see the dynamics of each compartments in (1) as a function of time and get Fig 12, which shows the number of infected individuals and mosquitoes decreases through time. Fig 13 shows the effect of temperature on the basic reproduction number, that is, the value starts to increase from 0 (at $15{\;}^{\circ }\text{C}$) to the maximum *R*_0_ = 10.51 which occurs at $T=26.1616{\;}^{\circ }\text{C}$ then it starts to decrease after $26.1616{\;}^{\circ }\text{C}$ and is almost zero at $32{\;}^{\circ }\text{C}$. This tells us the maximum severity of the infection occurs at temperature $26.1616{\;}^{\circ }\text{C}$ and hence peoples living in such areas are at a higher risk of the disease. With the same values of parameters and initial conditions above, the dynamics of each compartment in the model is shown in Fig 14, with different temperature values; for $T=15{\;}^{\circ }\text{C}$ and $T=32{\;}^{\circ }\text{C}$, which are the minimum and maximum temperatures at which all the temperature dependent parameters stated in Sect 3 are positive, for $T=26.1616{\;}^{\circ }\text{C}$ which is the temperature at which maximum *R*_0_ occurs and for $T=19{\;}^{\circ }\text{C}$ and $29{\;}^{\circ }\text{C}$, and hence obtained that the number of infected human hosts is maximum for $T=26.1616{\;}^{\circ }\text{C}$ and is minimum for $T=15{\;}^{\circ }\text{C}$.

**Fig 15:**
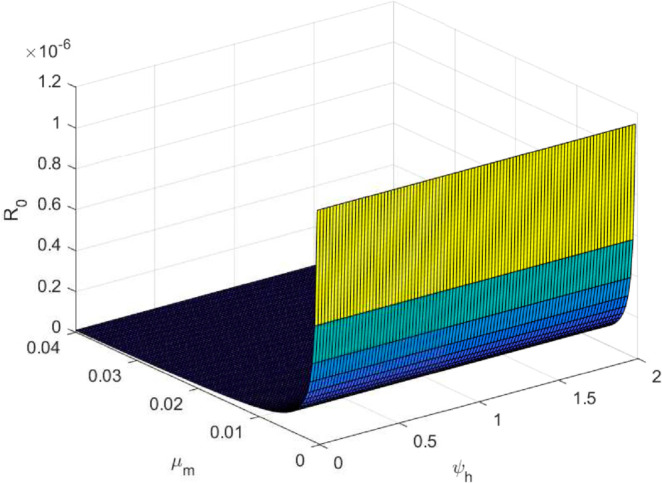
Sensitivity of mortality rate of mosquitoes and vaccination rate on *R*_0_.

**Fig 16:**
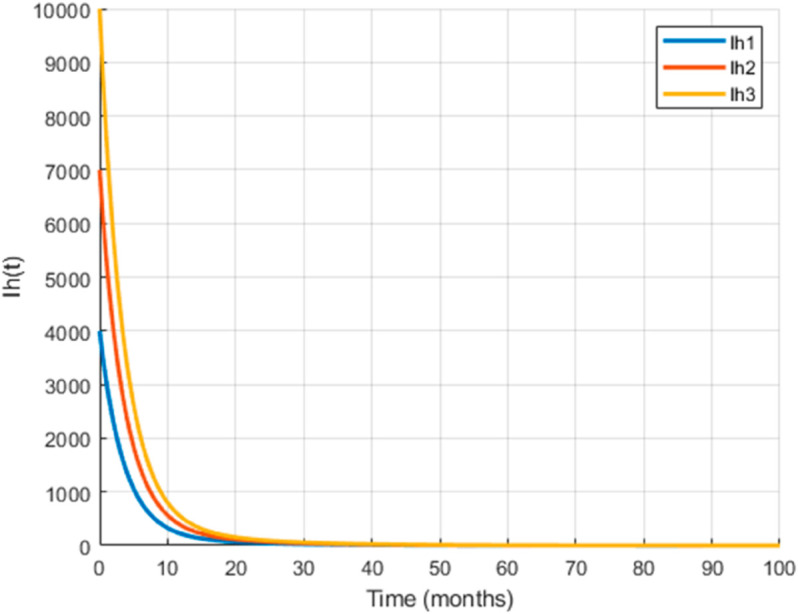
The infected population declines to zero over time for model (1) with the reproduction number $R_{0}≊0.0012<1$ indicating that the disease cannot persist and the DFE is globally stable under this threshold.

**Fig 17:**
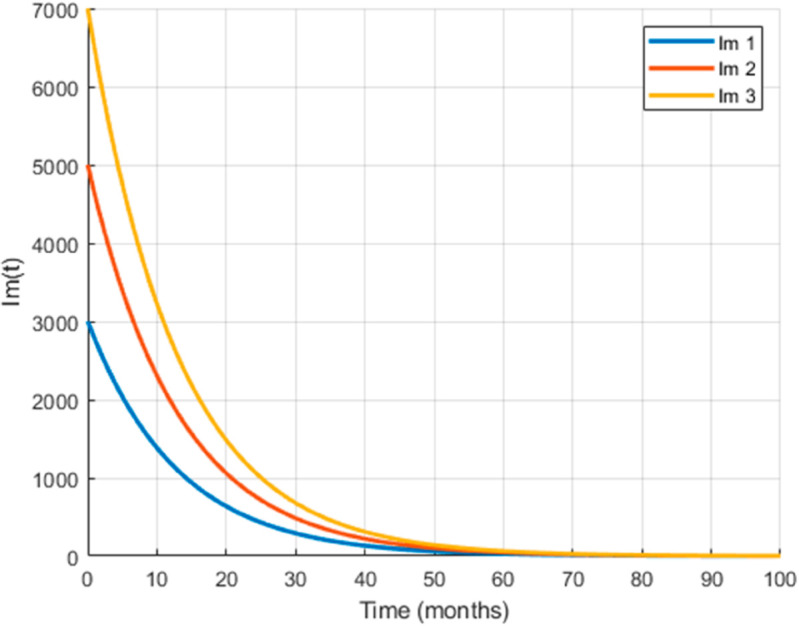
The infected population declines to zero over time for model (1) with the reproduction number $R_{0}≊0.0012<1$ indicating that the disease cannot persist and the DFE is globally stable under this threshold.

**Fig 18:**
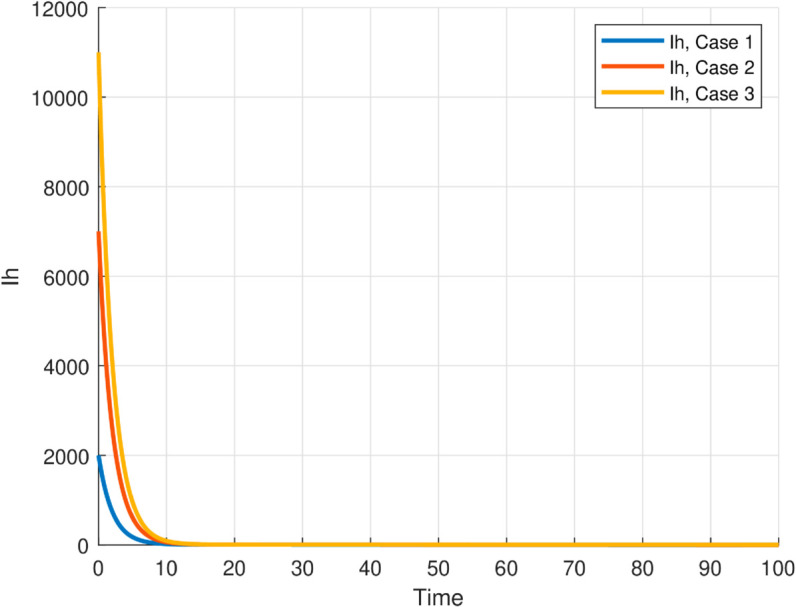
All trajectories, regardless of initial conditions, converge to the same endemic steady state, confirming the global asymptotic stability of EE for model (1) with the reproduction number $R_{0}≊5.9226>1$.

**Fig 19:**
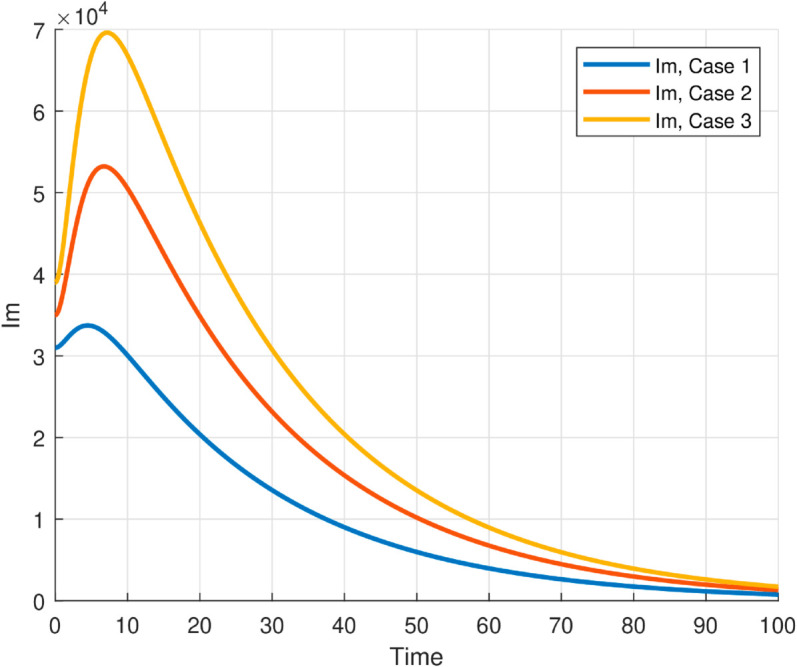
All trajectories, regardless of initial conditions, converge to the same endemic steady state, confirming the global asymptotic stability of EE for model (1) with the reproduction number $R_{0}≊5.9226>1$.

The maximum number of infected mosquitoes occurs for *T* = 26.1616 ∘C too. For $T=15{\;}^{\circ }\text{C}$, the amount of immature mosquitoes (*L*(*t*)) is negative which shows immature mosquitoes doesn’t exist at this temperature. We show that mosquito mortality rate and vaccination rate have negative relation with the basic reproduction number *R*_0_ and this shows, to decrease the transmission of the disease, increasing vaccination rate and mortality rate of mosquitoes is vital, and is shown in Fig 15. In Figs 16 and 17, the solution curves *I*_*h*_(*t*) and *I*_*m*_(*t*) converges to zero from different initial conditions, that shows the global asymptotical stability of diseases free equilibrium, which is also shown theoretically in theorem 4.2 before. As shown in theorem 4.6, Figs 18 and 19 shows the global asymptotical stability of the endemic equilibrium for *R*_0_ = 5.9226>1. To see the effect of the introduced control mechanisms, we solve the control system containing eight equations with their boundary conditions numerically. First we give starting values for the control variables made. The state variables are solved forward in-time using the control dynamics 14 and the given starting values. The values of the state variables obtained are used for computing the solutions for the adjoint Eqs 18 together with the given final conditions. We solved these backward in-time, using fourth order Runge-kutta method. The solutions of the state and adjoint variables are then used to update the control, and then until there is an adequate convergence between control values, the current state, and adjoint, these process is repeated. By using the same parameter values as in Figs 18, 19, and using $D_{1}=10,\;D_{2}=300$ and *D*_3_ = 200 to balance the population in the objective functional and assuming that all the proposed control mechanisms are not 100% effective and hence taking the upper bounds of each control mechanisms $c_{1};\;c_{2},\;and\;c_{3}$, respectively as 0.65,0.85,and0.88, we show the effect of the control mechanisms numerically.

## 7 Discussions and conclusions

**Fig 20:**
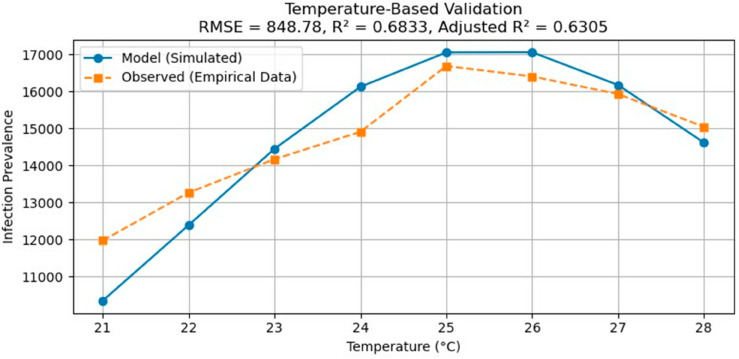
Comparison between model-predicted number of malaria infections and observed infection prevalence data from the Jimma zone as a function of temperature.

In this paper, we present a temperature-dependent, two-class-age-structured mathematical model of malaria, incorporating three optimal control strategies to assess the impact of temperature and control mechanisms on the transmission dynamics of malaria. By utilizing real-world data, several parameters were calibrated as functions of temperature. Our comprehensive analysis of the model encompasses the existence and positivity of solutions, the presence of equilibrium points, asymptotic smoothness, and the influences of temperature and control interventions on the transmission dynamics. To address the applicability and robustness of the temperature-dependent parameters in our malaria model, we performed an empirical validation using six years of observed infection prevalence data from Malaria incidence in Jimma zone, southwest of Ethiopia (2018 to 2023), which spans a range of temperature variations. Since the observed prevalence data were not age-structured, while our model incorporates an explicit age structure, we aggregated the age range from 0 to 60 years during simulation to ensure comparability. The model output (predicted number of infections as a function of temperature) was plotted alongside the empirical data (observed prevalence) for direct visual and quantitative comparison shown in Fig (20). Statistical measures showed a coefficient of determination ${\text{R}}^{2}$ of 0.68 and an adjusted ${\text{R}}^{2}$ of 0.63, indicating a reasonably strong association between model predictions and observed patterns across the temperature range. Although the absence of age-stratified empirical data introduces some limitation to the precision of validation, the results demonstrate that the model captures key trends in temperature-dependent malaria transmission. These findings support the applicability of the model across varying environmental conditions, and suggest robustness of the temperature-dependent parameters when applied to field data.

**Fig 21:**
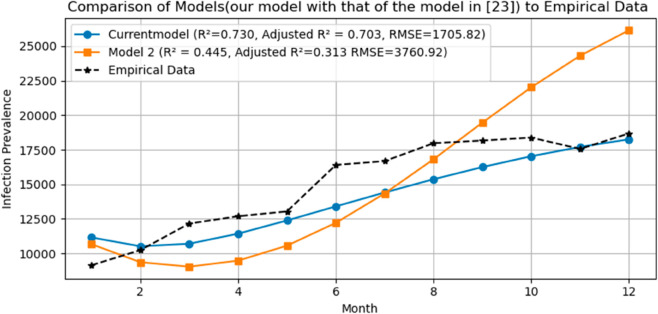
Direct comparison of model predictions from the present model and an existing literature model against observed malaria infection prevalence data from the Jimma zone.

**Fig 22:**
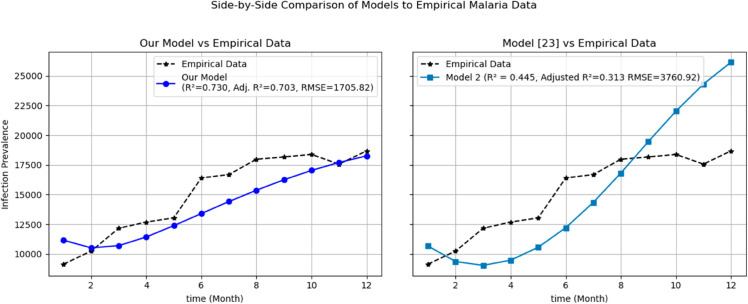
Side-by-side comparison of monthly infection prevalence predictions from our proposed model and the existing model in [23] against empirical data from the Jimma zone.

To further assess the predictive power and practical value of our model, we compared its outcomes against an existing age-structured malaria model from the literature [23]. Using the same empirical dataset (six years of infection prevalence data from the Jimma zone), both models were simulated and their outputs compared directly to the observed data. Statistical performance metrics including the coefficient of determination ${\text{R}}^{2}$, root mean squared error (RMSE), and adjusted ${\text{R}}^{2}$ were calculated for both models (Fig 21). Our model achieved an ${\text{R}}^{2}$ of 0.730 and an adjusted ${\text{R}}^{2}$ of 0.703, compared to the existing model’s ${\text{R}}^{2}$ of 0.445 and adjusted ${\text{R}}^{2}$ of 0.313. Similarly, our model achieved a lower RMSE (1705.82 vs. 3760.92). These results demonstrate that our model provides improved predictive accuracy and better captures the observed temperature-dependent variation in malaria transmission, thereby offering enhanced utility for prediction and control strategies compared to existing approaches. From Fig 22, the left side figure shows the fit of our age-structured malaria model, while the right side one presents the predictions of the model from [23], both compared to six years of observed monthly infection prevalence data.

**Fig 23:**
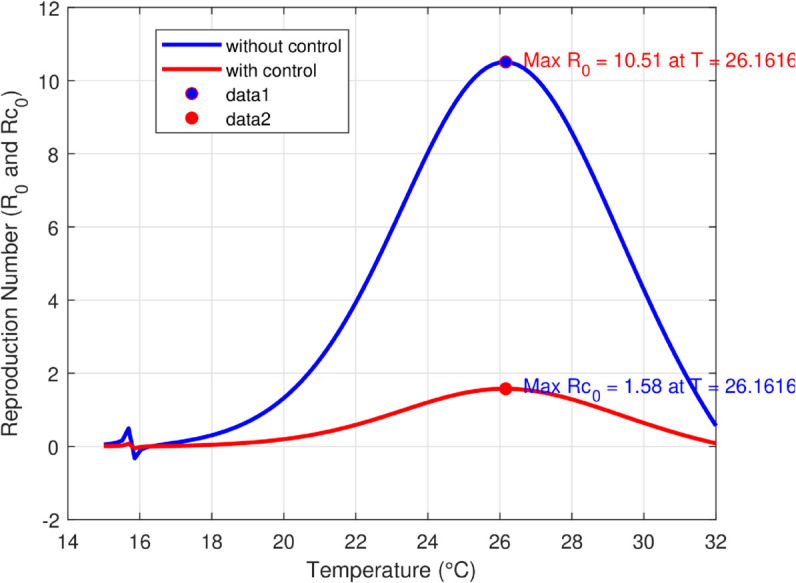
The figure shows that *R*_0_ decreases significantly under control strategies, particularly at temperatures near the peak transmission range, indicating the effectiveness of control mechanisms applied.

**Fig 24:**
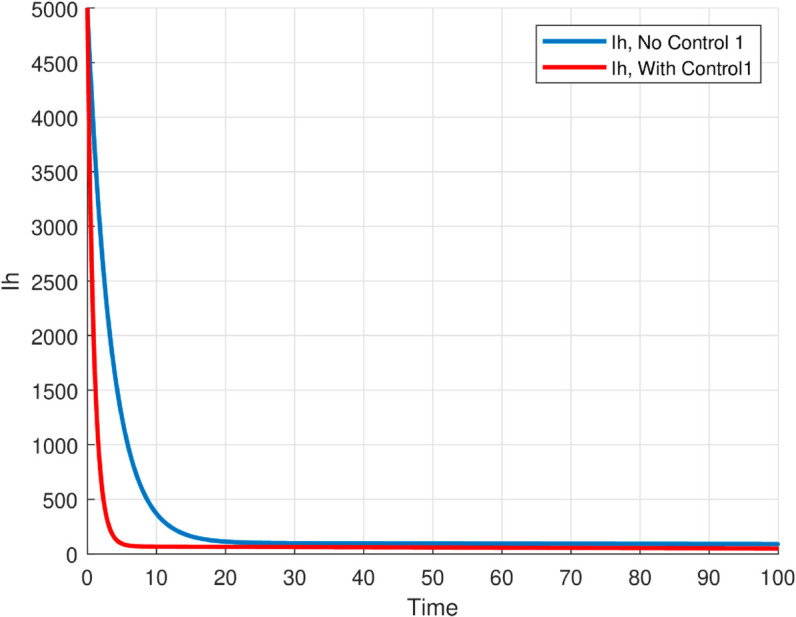
Time evolution of infected human and mosquito populations under optimal control compared to the uncontrolled case. The results demonstrate that applying control strategies significantly reduces infection levels in both populations over time, highlighting the effectiveness of the interventions in mitigating malaria transmission.

**Fig 25:**
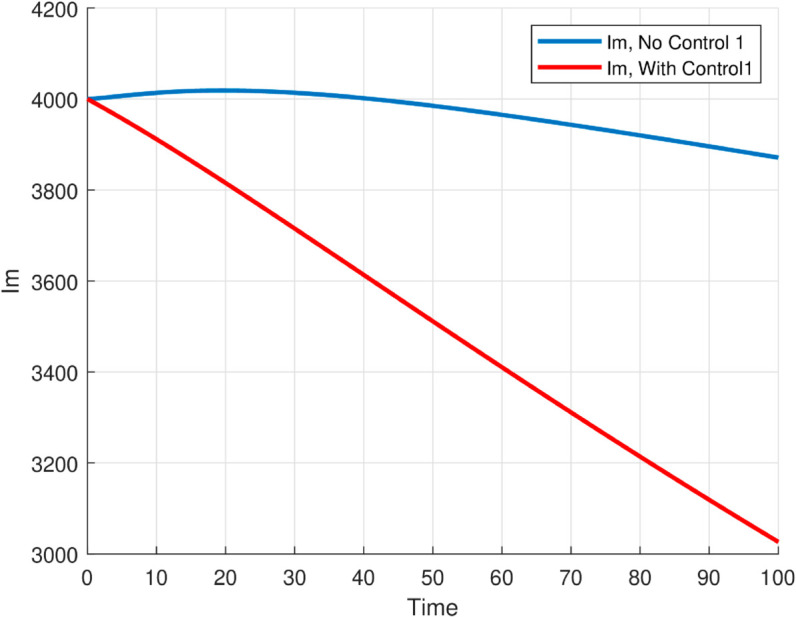
Time evolution of infected human and mosquito populations under optimal control compared to the uncontrolled case. The results demonstrate that applying control strategies significantly reduces infection levels in both populations over time, highlighting the effectiveness of the interventions in mitigating malaria transmission.

**Fig 26:**
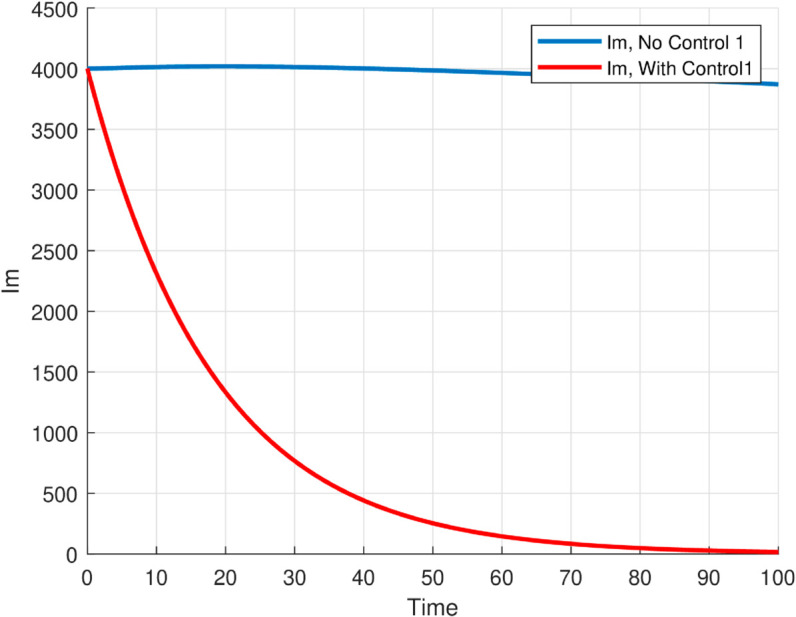
The solution (infected humans and mosquitoes) of optimal control problem, with and without controls with $c_{1}\neq 0,c_{2}\neq 0$ and *c*_3_ = 0 for the first graph and $c_{1},c_{3}\neq 0$ and *c*_2_ = 0 for the second graph.

**Fig 27:**
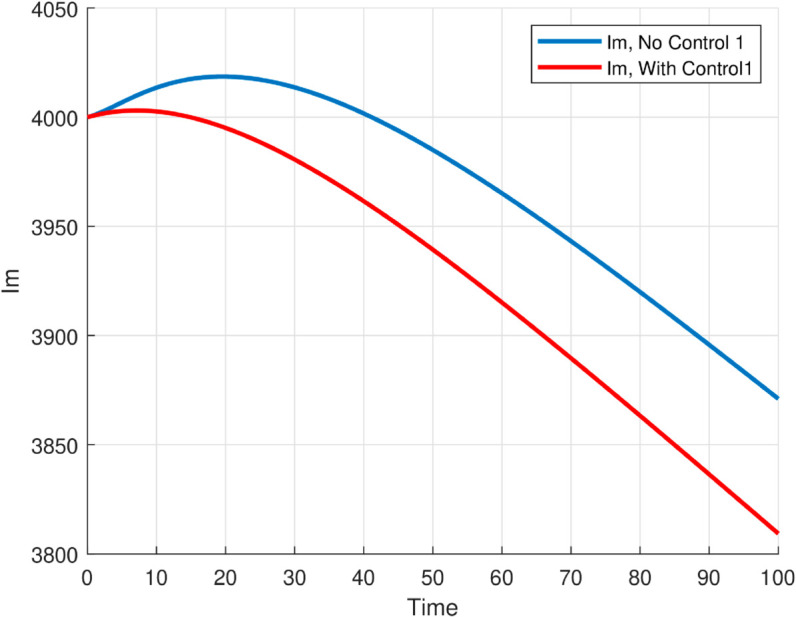
The solution (infected humans and mosquitoes) of optimal control problem, with and without controls with $c_{1}\neq 0,c_{2}\neq 0$ and *c*_3_ = 0 for the first graph and $c_{1},c_{3}\neq 0$ and *c*_2_ = 0 for the second graph.

In Fig 23, the basic reproduction number as the function of temperature with and without control mechanisms is shown, and hence get maximum *R*_0_ = 1.58 with control mechanisms, whereas maximum *R*_0_ = 10.51 in the absence of controls. The solution (infected human hosts and mosquitoes) of optimal control problem is shown in by red curve in Figs 24, 25 and the blue curve shows the solution without control mechanisms. These shows that the number of infected humans and mosquito vector reduced significantly under the influence of the stated control mechanisms. We can also observe from Figs 26 and 27 that one should use all the three control mechanisms in order to decrease severity of the infection significantly and the control mechanism *c*_2_(*t*)(which includes larvacide and adulticide) has more effect in order to decrease the number of infected mosquitoes. Basic reproduction number *R*_0_, derived which serves as a critical threshold for determining disease persistence and extinction. The findings indicate that the disease will disappear when *R*_0_<1, and the disease-free equilibrium is globally asymptotically stable. Conversely, when *R*_0_>1, the disease persists, and the endemic steady state exists, demonstrating global asymptotic stability. In reference [24], the authors illustrate how temperature-dependent parameters negatively affect the survival of both immature and adult mosquitoes at temperature levels below $16{\;}^{\circ }\text{C}$ and above $32{\;}^{\circ }\text{C}$, thereby influencing the transmission dynamics of malaria. Similarly, our results corroborate this finding; as illustrated in Fig 14, at a temperature of $15{\;}^{\circ }\text{C}$, the immature stages of mosquitoes are reduced to non-existent, indicating that no immature mosquitoes survive at this temperature. So, peoples who are living in this domain of temperature are at lower risk of the infection.

The temperature-dependent parameters in our model, are fitted using polynomial functions based on empirical data within the range $T\in [15^{\circ }C,32^{\circ }C]$. This temperature window corresponds to the biologically relevant interval for malaria transmission and mosquito development. Outside this range, such as the number of immature mosquitoes dropping below zero as shown in Fig 14, reflecting unviable environmental conditions for malaria transmission. Therefore, the polynomial fits are not extrapolated beyond $\left[15^{\circ }C,32^{\circ }C\right]$, and the model is only defined and interpreted within this temperature range. This restriction preserves the biological realism of the model and ensures that simulations remain consistent with known mosquito ecology and malaria dynamics.

Mordecai *et al*. [28] demonstrated that the optimal temperature for malaria transmission is $25{\;}^{\circ }\text{C}$, with transmission decreasing significantly above $28{\;}^{\circ }\text{C}$. Similarly, Agusto *et al*. [29] identified the favorable temperature range for mosquito growth across 67 sub-Saharan cities in Africa as $16{\;}^{\circ }\text{C}$ to $28{\;}^{\circ }\text{C}$. In this paper, we further illustrate the significant impact of temperature on malaria transmission dynamics by analyzing its effect on the basic reproduction number *R*_0_. Specifically, *R*_0_ increases from zero at $T=15{\;}^{\circ }\text{C}$ to a peak value of *R*_0_ = 10.15 at $T=26.16{\;}^{\circ }\text{C}$, before declining back to zero beyond $T=32{\;}^{\circ }\text{C}$. This indicates that the maximum severity of infection occurs at $T=26.16{\;}^{\circ }\text{C}$, placing populations in regions with such temperatures are at the highest risk of malaria transmission. By extending the model to incorporate an optimal control framework with three control strategies $\left(c_{1},\;c_{2},\;and\;c_{3}\right)$, we observed a substantial reduction in malaria transmission dynamics, as shown in Figs 23, 24 and 25. Moreover, as illustrated in Figs 26 and 27, combining all three control mechanisms is the most effective strategy for reducing the number of infected humans and mosquitoes, thereby significantly decreasing the overall malaria burden. These findings highlight the model’s enhanced capability to capture temperature-driven variations in malaria transmission and its potential utility for informing prediction and control strategies, particularly under changing environmental conditions. Future work will focus on extending the validation to other geographical regions and incorporating age-specific empirical data as it available.

### Future work

Future extensions of this study will focus on incorporating additional climatic factors such as humidity and rainfall, which play a significant role in mosquito breeding, survival, and malaria transmission dynamics. Including these variables will improve the model’s accuracy and ecological realism, especially in regions with seasonal transmission patterns. We also aim to integrate the model with real-time environmental data streams (e.g., satellite-based temperature, humidity, and rainfall datasets) to enable dynamic prediction of malaria risk. Furthermore, linking the model to public health decision-support systems can facilitate early warning alerts, resource optimization, and targeted vector control interventions. Such integration would significantly enhance the model’s operational value for malaria surveillance and control programs.

## Figures and Tables

**Table 5:**
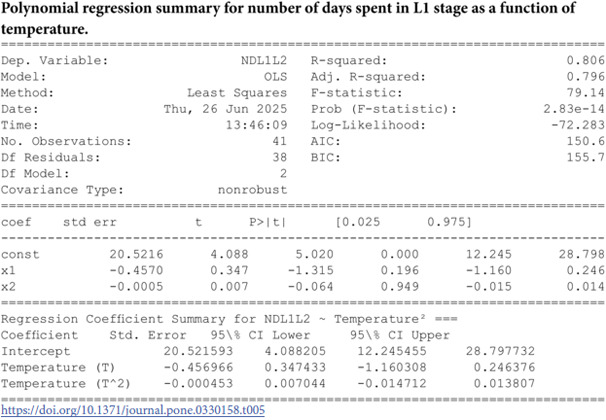
Polynomial regression summary for number of days spent in L1 stage as a function of temperature.

**Table 6:**
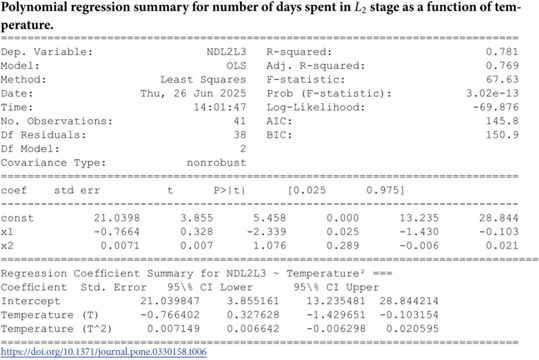
Polynomial regression summary for number of days spent in *L*_2_ stage as a function of temperature.

**Table 7:**
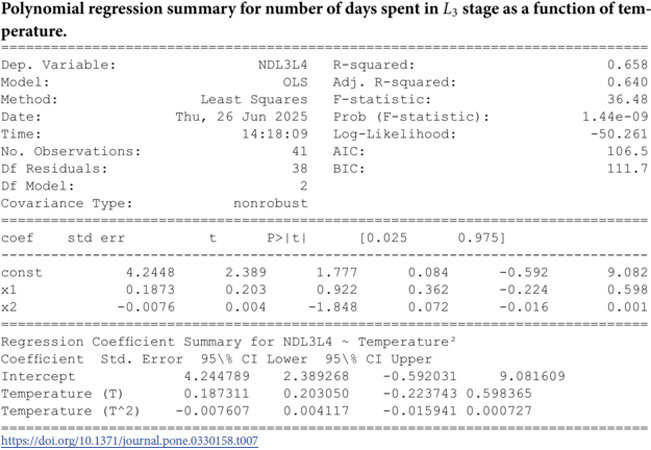
Polynomial regression summary for number of days spent in *L*_3_ stage as a function of temperature.

**Table 8:**
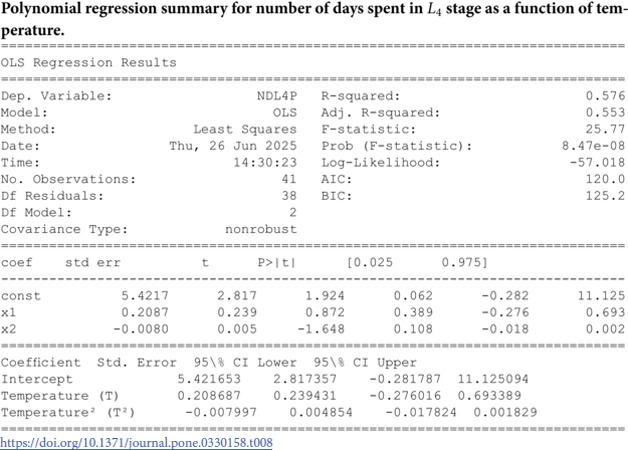
Polynomial regression summary for number of days spent in *L*_4_ stage as a function of temperature.

**Table 9:**
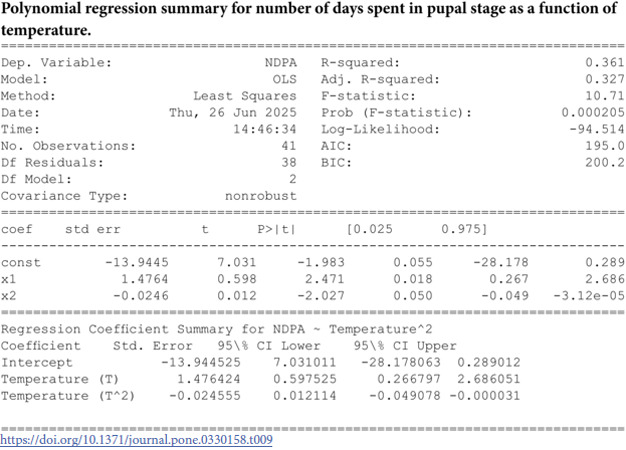
Polynomial regression summary for number of days spent in pupal stage as a function of temperature.

**Table 10:**
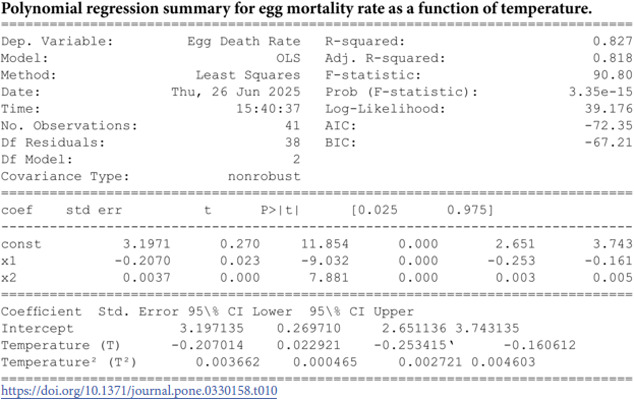
Polynomial regression summary for egg mortality rate as a function of temperature.

**Table 11:**
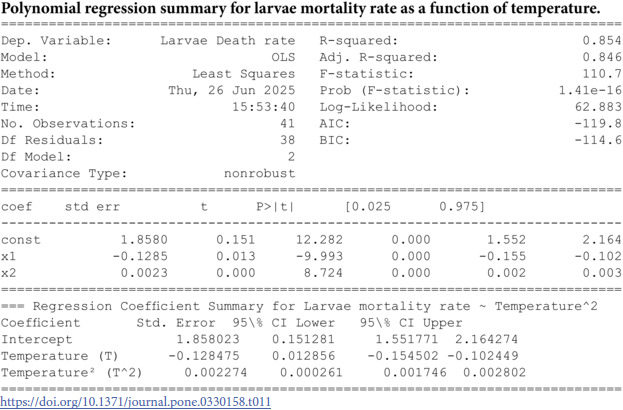
Polynomial regression summary for larvae mortality rate as a function of temperature.

**Table 12:**
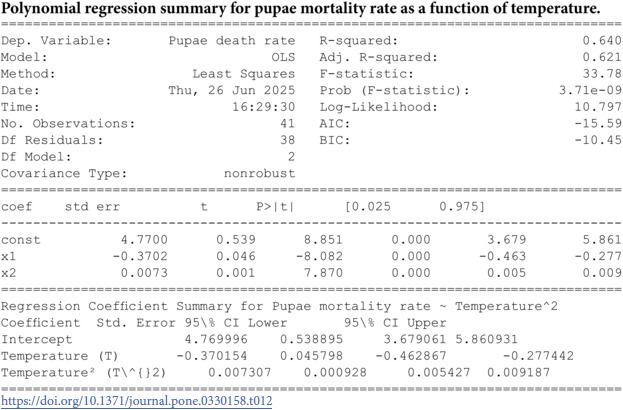
Polynomial regression summary for pupae mortality rate as a function of temperature.

**Table 13:**
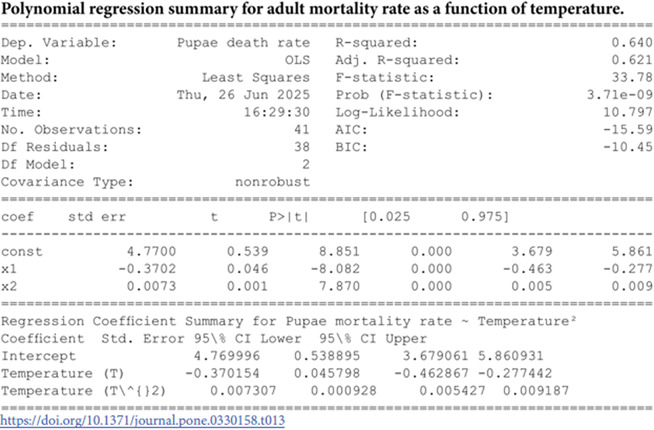
Polynomial regression summary for adult mortality rate as a function of temperature.

## Appendix 2: Proof of theorem 4.2

*Proof*: Define $\rho_{1}(a)=\int_{0}^{\infty }\xi (s)e^{-\int_{a}^{s}k_{1}(\theta )d\theta }ds,\;\rho_{2}(b)=\int_{0}^{\infty }\gamma (s)e^{-\int_{b}^{s}k_{2}(\theta )d\theta }ds$.

Here, $\rho_{1}(0)=\eta_{1}$ and $\rho_{2}(0)=\eta_{2}$. In addition $\rho_{1}(a)$ and $\rho_{2}(b)$ are bounded and satisfies;

$\rho_{1}^{\prime }(a)=\rho_{1}(a)k_{1}(a)-\xi (a)$ and $\rho_{2}^{\prime }(b)=\rho_{2}(b)k_{2}(b)-\gamma (b)$. Now define the Lyapunov functional;

$$ℒ=ℒ(S_{h},\;V,\;I_{h},\;R,\;L,\;S_{m},\;E_{m},\;I_{m})={ℒ}_{1}+{ℒ}_{2}+{ℒ}_{3},\;where,$$

$${ℒ}_{1}=\frac{1}{S_{h0}}\left(S_{h}-S_{h0}-S_{h0}I_{h}\left(\frac{S_{h}}{S_{h0}}\right)\right)+\frac{1}{S_{h0}S_{m0}}I_{h},\;\;\;\;\;\;\;\;\;\;\;\;\;\;\;\;\;\;\;\;\;\;\;\;\;\;\;\;\;\;\;\;\;\;\;\;\;\;\;\;\;\;\;\;\;\;\;\;\;\;\;\;\;\;\;\;\;\;\;\;\;\;\;\;\;\;\;\;{ℒ}_{2}=\frac{1}{S_{h0}S_{m0}}\int_{0}^{\infty }\rho_{1}(a)\left(V(a,t)-V(a)-V(a)I_{h}\left(\frac{V(a,t)}{V(a)}\right)\right)da+\frac{1}{S_{h0}S_{m0}}\int_{0}^{\infty }\rho_{2}(b)R(b,t)db,\;{ℒ}_{3}=\frac{\lambda_{mh}b}{\omega N_{h}}\left[\frac{S_{m}}{S_{m0}}-1-ln\left(\frac{S_{m}}{S_{m0}}\right)\right]+\frac{\lambda_{mh}b}{\omega N_{h}}\frac{I_{m}}{I_{m0}}.\;\;\;\;\;\;\;\;\;\;\;\;\;\;\;\;\;\;\;\;\;\;\;\;\;\;\;\;\;\;\;\;\;\;\;\;\;\;\;\;\;\;\;\;\;\;\;\;\;\;\;\;\;\;\;\;\;\;\;\;\;\;\;\;\;\;\;\;\;\;\;\;\;\;$$

Here, $ℒ=ℒ(S_{h},\;V,\;I_{h},\;R,\;L,\;S_{m},\;E_{m},\;I_{m})\geq 0$ and $ℒ(x)=0\;⟺\;x=(S_{h0},\;0,\;0,\;0,\;L_{0},\;S_{m0},\;0,\;0)$. Now, we calculate derivatives of ${ℒ}_{1},\;{ℒ}_{2},\;and\;{ℒ}_{3}$ with respect to time along the solution of the model (1). That is;

$$\frac{d{ℒ}_{1}}{dt}=\frac{d}{dt}\left[\frac{1}{S_{h0}}\left(S_{h}-S_{h0}-S_{h0}I_{h}\left(\frac{S_{h}}{S_{h0}}\right)\right)+\frac{1}{S_{h0}S_{m0}}I_{h}\right]=\frac{1}{S_{h0}}\left[\frac{dS_{h}}{dt}-\frac{S_{h0}}{S_{h}}\frac{dS_{h}}{dt}\right]$$

$$+\frac{1}{S_{h0}S_{m0}}\frac{dI_{h}}{dt}=\frac{1}{S_{h0}}\left[\wedge_{h}-(\psi_{h}+\mu_{h})S_{h}-\frac{\lambda_{mh}b}{N_{h}}I_{m}S_{h}+\int_{0}^{\infty }\xi (a)V(a,t)da\right]$$

$$+\frac{1}{S_{h0}S_{m0}}\left[\frac{\lambda_{mh}b}{N_{h}}I_{m}S_{h}-(\beta_{1}+\mu_{h}+\nu )I_{h}+\int_{0}^{\infty }\gamma (b)R(b,t)db\right]$$

$$=\frac{1}{S_{h0}}\left[(\mu_{h}+\psi_{h})S_{h0}-\int_{0}^{\infty }\rho_{1}(a)V_{0}(a)da-(\mu_{h}+\psi_{h})S_{h}-\frac{\lambda_{mh}b}{N_{h}}I_{m}S_{h}+\int_{0}^{\infty }\xi (a)V(a,t)da\right]$$

$$+\frac{1}{S_{h0}S_{m0}}\left[\frac{\lambda_{mh}b}{N_{h}}I_{m}S_{h}-(\beta_{1}+\mu_{h}+\nu )I_{h}+\int_{0}^{\infty }\gamma (b)R(b,t)db\right]$$

$$=\left[(\mu_{h}+\psi_{h})\left(1-\frac{S_{h}}{S_{h0}}\right)-\frac{\lambda_{mh}b}{N_{h}}I_{m}\frac{S_{h}}{S_{h0}}+\frac{1}{S_{h0}}\left(\int_{0}^{\infty }\xi (a)V(a,t)da-\int_{0}^{\infty }\rho_{1}(a)V_{0}(a)da\right)\right]$$

$$+\frac{\lambda_{mh}b}{N_{h}}\frac{I_{m}}{S_{m0}}\frac{S_{h}}{S_{h0}}-(\beta_{1}+\mu_{h}+\nu )\frac{I_{h}}{S_{h0}S_{m0}}+\frac{1}{S_{h0}S_{m0}}\int_{0}^{\infty }\gamma (b)R(b,t)db.$$

$$=\left[(\mu_{h}+\psi_{h})\left(1-\frac{S_{h}}{S_{h0}}\right)-\frac{\lambda_{mh}b}{N_{h}}I_{m}\frac{S_{h}}{S_{h0}}+\frac{1}{S_{h0}}\int_{0}^{\infty }(\xi (a)V(a,t)-\rho_{1}(a)V_{0}(a))da\right]$$

$$+\frac{\lambda_{mh}b}{N_{h}}\frac{I_{m}}{S_{m0}}\frac{S_{h}}{S_{h0}}-(\beta_{1}+\mu_{h}+\nu )\frac{I_{h}}{S_{h0}S_{m0}}+\frac{1}{S_{h0}S_{m0}}\int_{0}^{\infty }\gamma (b)R(b,t)db.$$

$$=\left[(\mu_{h}+\psi_{h})\left(1-\frac{S_{h}}{S_{h0}}\right)+\frac{\lambda_{mh}b}{N_{h}}I_{m}\frac{S_{h}}{S_{h0}}\left(\frac{1}{S_{m0}}-1\right)+\frac{1}{S_{h0}}\int_{0}^{\infty }(\xi (a)V(a,t)-\rho_{1}(a)V_{0}(a))da\right]$$

$$-(\beta_{1}+\mu_{h}+\nu )\frac{I_{h}}{S_{h0}S_{m0}}+\frac{1}{S_{h0}S_{m0}}\int_{0}^{\infty }\gamma (b)R(b,t)db.$$

$$\frac{d{ℒ}_{2}}{dt}=\frac{d}{dt}\left[\frac{1}{S_{h0}S_{m0}}\int_{0}^{\infty }\rho_{1}(a)\left(V(a,t)-V(a)-V(a)I_{h}\left(\frac{V(a,t)}{V(a)}\right)\right)da+\frac{1}{S_{h0}S_{m0}}\int_{0}^{\infty }\rho_{2}(b)R(b,t)db\right].$$

$$=\frac{1}{S_{h0}S_{m0}}\int_{0}^{\infty }\rho_{1}(a)\frac{d}{dt}\left[V(a,t)-V(a)-V(a)ln\left(\frac{V(a,t)}{V_{0}(a)}\right)\right]da+\frac{1}{S_{h0}S_{m0}}\int_{0}^{\infty }\rho_{2}(b)\frac{d}{dt}\left(R(b,t)\right)db.$$

$$=\frac{1}{S_{h0}S_{m0}}\int_{0}^{\infty }\rho_{1}(a)\left(\frac{\partial V}{\partial t}-V_{0}(a)\frac{\partial V}{\partial t}\right)da+\frac{1}{S_{h0}S_{m0}}\int_{0}^{\infty }\rho_{2}(b)\frac{\partial R(b,t)}{\partial t}db.$$

$$=\frac{1}{S_{h0}S_{m0}}\int_{0}^{\infty }\rho_{1}(a)(1-V_{0}(a))\left[-\xi (a)V(a,t)-\mu_{h}V(a,t)-\frac{\partial V(a,t)}{\partial a}\right]da$$

$$+\frac{1}{S_{h0}S_{m0}}\int_{0}^{\infty }\rho_{2}(b)\left(-\mu_{h}R-\gamma (b)R-\frac{\partial R(b,t)}{\partial b}\right)db.$$

$$=\frac{1}{S_{h0}S_{m0}}\int_{0}^{\infty }\rho_{1}(a)(1-V_{0}(a))\left(-(\xi (a)+\mu_{h})V(a,t)-\frac{\partial V(a,t)}{\partial a}\right)da$$

$$+\frac{1}{S_{h0}S_{m0}}\int_{0}^{\infty }\rho_{2}(b)\left(-(\mu_{h}+\gamma (b))R(b,t)-\frac{\partial R(b,t)}{\partial b}\right)db+\frac{\eta_{1}\psi_{h}}{S_{m0}}\left[S_{h}-S_{h0}-S_{h0}ln\left(\frac{S_{h}}{S_{h0}}\right)\right]$$

$$+\eta_{2}\beta_{1}\frac{I_{h}}{S_{h0}S_{m0}}-\frac{1}{S_{h0}S_{m0}}\int_{0}^{\infty }\gamma (b)R(b,t)db-\frac{1}{S_{h0}S_{m0}}\int_{0}^{\infty }\xi (a)V_{0}(a)$$

$$\left(V(a,t)-V_{0}(a)-V_{0}(a)ln\left(\frac{V(a,t)}{V_{0}(a)}\right)\right)da.\frac{d{ℒ}_{3}}{dt}$$

$$=\frac{d}{dt}\left[\frac{\lambda_{mh}b}{\omega N_{h}}\left[\frac{S_{m}}{S_{m0}}-1-ln\left(\frac{S_{m}}{S_{m0}}\right)\right]+\frac{\lambda_{mh}b}{\omega N_{h}}\frac{I_{m}}{I_{m0}}\right]$$

$$=\frac{\lambda_{mh}b}{\omega N_{h}}\left[\frac{1}{S_{m0}}\frac{dS_{m}}{dt}-\frac{1}{S_{m}}\frac{dS_{m}}{dt}\right]+\frac{\lambda_{mh}b}{\omega N_{h}}\frac{1}{S_{m0}}\frac{dS_{m}}{dt}=\frac{\lambda_{mh}b}{\omega N_{h}}\left(\frac{1}{S_{m0}}-\frac{1}{S_{m}}\right)\frac{dS_{m}}{dt}+\frac{\lambda_{mh}b}{\omega N_{h}S_{m0}}\frac{dI_{m}}{dt}.$$

$$=\frac{\lambda_{mh}b}{\omega N_{h}}\left(\frac{1}{S_{m0}}-\frac{1}{S_{m}}\right)\left(\sigma_{m}L-\omega S_{m}-\frac{\lambda_{hm}b}{N_{h}}I_{h}S_{m}\right)+\frac{\lambda_{mh}b}{\omega N_{h}S_{m0}}(\beta_{2}E_{m}-\omega I_{m}).$$

$$But,\;\frac{dℒ}{dt}=\frac{d{ℒ}_{1}}{dt}+\frac{d{ℒ}_{2}}{dt}+\frac{d{ℒ}_{3}}{dt}.$$

$$=-\left(\frac{\mu_{h}+\psi_{h}(1-\eta_{1})}{S_{m0}}\right)\left[\frac{S_{h}}{S_{h0}}-1-ln\left(\frac{S_{h}}{S_{h0}}\right)+\frac{S_{h0}}{S_{h}}-1-ln\left(\frac{S_{h0}}{S_{h}}\right)\right]$$

$$-\frac{\lambda_{mh}b}{N_{h}}\left[\frac{S_{m0}}{S_{m}}-1-ln\left(\frac{S_{m0}}{S_{m}}\right)=\frac{S_{m}}{S_{m0}}-1-ln\left(\frac{S_{m}}{S_{m0}}\right)\right]$$

$$-\frac{\psi_{h}}{S_{m0}}\left[\frac{V(a,t)}{V_{0}(a)}\frac{S_{h0}}{S_{h}}-1-ln\left(\frac{V(a,t)}{V_{0}(a)}\frac{S_{h0}}{S_{h}}\right)\right]+(R_{0}-1)I_{h}.$$

Therefore, *R*_0_<1 implies that $\frac{dℒ}{dt}\leq 0$. In addition $\frac{dℒ}{dt}=0$ only if $S_{h}(t)=S_{h0},\;V(a,t)=V_{0}(a),\;I_{h}(t)=0,\;R(b,t)=0,\;S_{m}(t)=S_{m0},\;L(t)=L_{0},\;E_{m}(t)=I_{m}(t)=0$, simultaneously. Therefore, the disease free equilibrium is globally asymptotically stable by the Lyapunov-Lasalle’s principle. □

For $y=(y_{1},\;y_{2},\;y_{3},\cdots ,y_{8})\in 𝒴$, with the reasonable modifications of the proofs of theorem 2.1 and lemma 2.2 in [12], we can prove the existence and non-negativeness of solutions to our model 1.

## Appendix 3: Proof of theorem 4.6

*Proof*: Suppose $E^{*}=\left(S_{h}^{*},\;V^{*},\;I_{h}^{*},\;R^{*},\;L^{*},\;S_{m}^{*},\;E_{m}^{*},\;I_{m}^{*}\right)$ be the endemic steady state of model (1). By theorem 4.5, $ℬ=\{E^{*}\}$. To construct a Lyapunov functional, let us define a function h:(0,∞)→R, such that h(x)=x−1−lnx. Hence, h(x)≥0 for all x∈(0,∞) and h(x)=0 if and only if *x* = 1.

Let $S_{h}(t),\;V(\cdot ,t),\;I_{h}(t),\;R(\cdot ,t),\;L(t),\;S_{m}(t),\;E_{m}(t),\;I_{m}(t)$ be a total trajectory in ℬ. Note that all $S_{h}(t),\;V(0,\;t),\;I_{h}(t),\;R(0,\;t),\;L(t),\;S_{m}(t),\;E_{m}(t),\;and\;I_{m}(t)$ are bounded above. Moreover, by theorem 4.5 above, they are also bounded away from zero. Therefore, there exists an $\epsilon_{0}>0$ such that $0\leq h(x)<\epsilon_{0}$ for

$$x=\frac{S_{h}(t)}{S_{h}^{*}(t)},\;\frac{V(0,\;t)}{V^{*}(0)},\;\frac{I_{h}(t)}{I_{h}^{*}(t)},\;\frac{R(0,\;t)}{R^{*}(0)},\;\frac{L(t)}{L^{*}},\;\frac{S_{m}(t)}{S_{m}^{*}(t)},\;\frac{E_{m}(t)}{E_{m}^{*}(t)},\;\frac{I_{m}(t)}{I_{m}^{*}(t)},\;\forall t\in R.\;Noting$$

$$\frac{V(a,\;t)}{V^{*}(a)}=\frac{V(0,\;t-a)}{V^{*}(0)}\;and\;\frac{R(b,\;t)}{R^{*}(b)}=\frac{R(0,\;t-b)}{R^{*}(0)},\;we\;have\;0\leq h\left(\frac{V(a,\;t)}{V^{*}(a)}\right)<\epsilon_{0}\;and\;0\leq \;h\left(\frac{R(b,\;t)}{R^{*}(b)}\right)<\epsilon_{0}$$

for all t∈R and $a,\;b\in R_{+}$. Now define a Lyapunov functional as $F=F_{1}+F_{2}+F_{3}+\cdots +F_{8}$, where, $F_{1}=h\left(\frac{S_{h}}{S_{h}^{*}}\right),\;F_{2}=\frac{1}{S_{h}^{*}}\int_{0}^{\infty }\rho_{1}(a)V^{*}(a)h\left(\frac{V(a,\;t)}{V^{*}(a)}\right)da,\;F_{3}=\frac{I_{h}^{*}}{S_{h}^{*}}h\left(\frac{I_{h}}{I_{h}^{*}}\right),\;F_{4}=\frac{1}{S_{h}^{*}}\int_{0}^{\infty }\rho_{2}(b)R^{*}(b)h\left(\frac{R(b,\;t)}{R^{*}(b)}\right)db,$

$F_{5}=h\left(\frac{L}{L^{*}}\right),\;F_{6}=\frac{b\beta_{2}S_{m}^{*}\frac{\lambda_{mh}}{N_{h}}}{(\beta_{2}+\omega )\omega }h\left(\frac{S_{m}}{S_{m}^{*}}\right),\;F_{7}=\frac{\lambda_{mh}b\beta_{2}}{N_{h}(\beta_{2}+\omega )\omega }h\left(\frac{E_{m}}{E_{m}^{*}}\right),\;and\;F_{8}=\frac{\lambda_{mh}b}{\omega N_{h}}h\left(\frac{I_{m}}{I_{m}^{*}}\right)$.

Calculating the derivatives of *F*_1_ using $\wedge_{h}=\left(\psi_{h}+\mu_{h}+\frac{\lambda_{mh}b\lambda_{hm}I_{m}^{*}\;}{N_{h}}\right)S_{h}^{*}+\int_{0}^{\infty }\xi (a)V^{*}(a)da$, we get

$$\frac{dF_{1}}{dt}=(\mu_{h}+\psi_{h})\left(2-\frac{S_{h}}{S_{h}^{*}}-\frac{S_{h}^{*}}{S_{h}}\right)+\frac{\lambda_{mh}b}{N_{h}}I_{m}^{*}\left(1-\frac{S_{h}I_{m}}{S_{h}^{*}I_{m}^{*}}-\frac{S_{h}^{*}V(a,\;t)}{S_{h}V^{*}(a)}-\frac{S_{h}^{*}}{S_{h}}+\frac{I_{m}}{I_{m}^{*}}\right)\;+\int_{0}^{\infty }\frac{\xi (a)V^{*}(a)}{S_{h}^{*}}\left[\frac{V(a,\;t)}{V^{*}(a)}-\frac{S_{h}^{*}V(a,\;t)}{S_{h}V^{*}(a)}+\frac{S_{h}^{*}}{S_{h}}+1\right]da.$$
(24)

Derivatives of *F*_2_ along the solutions of model (1) also satisfies;

$$\frac{dF_{2}}{dt}=-\frac{\rho_{1}(a)V^{*}(a)}{S_{h}^{*}}\left(\frac{V(a,\;t)}{V^{*}(a)}\right){|}_{a=\infty }+\eta_{1}\psi_{h}h\left(\frac{S_{h}}{S_{h}^{*}}\right)-\int_{0}^{\infty }\frac{\xi (a)V^{*}(a)}{S_{h}^{*}}h\left(\frac{V(a,\;t)}{V^{*}(a)}\right)da.$$
(25)

In the same way the derivatives of $F_{3},\;F_{4},\;F_{5},\cdots F_{8}$ are obtained as;

$$\frac{dF_{3}}{dt}=\frac{\lambda_{mh}b}{N_{h}}I_{m}^{*}\left[\frac{S_{h}I_{m}}{S_{h}^{*}I_{m}^{*}}-\frac{S_{h}I_{m}I_{h}^{*}}{S_{h}^{*}I_{m}^{*}I_{h}}-\frac{I_{h}}{I_{h}^{*}}+1\right]$$
(26)

$$+\int_{0}^{\infty }\frac{\gamma (b)}{S_{h}^{*}}\times R^{*}(b)\left(\frac{R(b,\;t)}{R^{*}(b)}-\frac{I_{h}^{*}R(b,\;t)}{I_{h}R^{*}(b)}-\frac{I_{h}}{I_{h}^{*}}+1\right)db.$$

$$\frac{dF_{4}}{dt}=-\frac{\rho_{2}(b)R^{*}(b)}{S_{h}^{*}}h\left(\frac{R(b,\;t)}{R^{*}(b)}{|}_{b=\infty }\right)+\eta_{2}\beta_{1}\frac{I_{h}^{*}}{I_{h}}h\left(\frac{I_{h}}{I_{h}^{*}}\right)-\int_{0}^{\infty }\frac{\gamma (b)R^{*}(b)}{S_{h}^{*}}\left(\frac{R(b,\;t)}{R^{*}(b)}\right)db.$$
(27)

$$\frac{dF_{5}}{dt}=\frac{1}{L}\left[(\omega_{+}\sigma_{m})L^{*}-(\omega_{l}+\sigma_{m})L\right].$$
(28)

$$\frac{dF_{6}}{dt}=\frac{\lambda_{mh}b\beta_{2}S_{m}^{*}}{N_{h}(\beta_{2}+\omega )}\left[\left(\frac{S_{m}^{*}}{S_{m}}-1\right)\omega +\frac{\lambda_{hm}bS_{m}^{*}I_{h}}{N_{h}}\left(\frac{I_{h}^{*}S_{m}^{*}}{I_{h}S_{m}}-1\right)\right].$$
(29)

$$\frac{dF_{7}}{dt}=\frac{\lambda_{mh}b\beta_{2}}{N_{h}\omega }\left[\frac{E_{m}^{*}}{E_{m}}-1\right]\;and$$
(30)

$$\frac{dF_{8}}{dt}=\frac{\lambda_{mh}b}{N_{h}}\left[\frac{I_{m}^{*}}{I_{m}}-1\right].$$
(31)

Summing up equations (4.10)–(4.17), we get

$$\frac{dF}{dt}=\frac{dF_{1}}{dt}+\frac{dF_{2}}{dt}+\frac{dF_{3}}{dt}+\frac{dF_{4}}{dt}+\frac{dF_{5}}{dt}+\frac{dF_{6}}{dt}+\frac{dF_{7}}{dt}+\frac{dF_{8}}{dt}.\;\;\;\;\;\;\;\;\;\;\;\;\;\;\;\;\;\;\;\;\;\;\;\;\;\;\;\;\;\;\;\;\;\;\;\;\;\;\;\;\;\;\;\;\;\;\;=-\left(\mu_{h}+\psi_{h}(1-\eta_{1})\right)\left[\frac{S_{h}}{S_{h}^{*}}-1+ln\left(\frac{S_{h}}{S_{h}^{*}}\right)+\frac{S_{h}^{*}}{S_{h}}-1+ln\left(\frac{S_{h}^{*}}{S_{h}}\right)\right]\;\;\;\;\;\;\;\;\;\;\;\;\;\;\;\;\;\;\;\;\;\;\;\;\;\;\;\;\;\;\;\;\;\;\;\;\;\;\;\;-\frac{\rho_{1}(a)V^{*}(a)}{S_{h}^{*}}\left[\frac{V(a,t)}{V^{*}(a)}-1+ln\left(\frac{V(a,\;t)}{V^{*}(a)}\right)\right]{|}_{a=\infty }-\psi_{h}\eta_{1}\left[\frac{S_{h}^{*}V(a,\;t)}{S_{h}V^{*}(a)}-1+ln\left(\frac{S_{h}^{*}V(a,\;t)}{S_{h}V^{*}(a)}\right)\right]\;-\frac{\rho_{2}(b)R^{*}(b)}{S_{h}^{*}}\left[\frac{R(b,\;t)}{R^{*}(b)}-1+ln\left(\frac{R(b,\;t)}{R^{*}(b)}\right)\right]{|}_{b=\infty }-K\eta_{2}\frac{I_{h}^{*}}{S_{h}^{*}}\left[\frac{I_{h}^{*}R(b,\;t)}{I_{h}R^{*}(b)}-1+ln\left(\frac{I_{h}^{*}R(b,\;t)}{I_{h}R^{*}(b)}\right)\right]\;-\frac{\lambda_{mh}b}{N_{h}}I_{m}^{*}\left[\frac{S_{h}^{*}}{S_{h}}-1+ln\left(\frac{S_{h}^{*}}{S_{h}}+(\omega_{l}+\sigma_{m})\left(\frac{L^{*}}{L}-1\right)\right)\right]\;\;\;\;\;\;\;\;\;\;\;\;\;\;\;\;\;\;\;\;\;\;\;\;\;\;\;\;\;\;\;\;\;\;\;\;\;\;\;\;\;\;\;\;\;\;\;\;\;\;\;\;\;\;\;\;\;-\frac{\lambda_{mh}b}{N_{h}}I_{m}^{*}\left[\frac{S_{h}I_{m}I_{h}^{*}}{S_{h}^{*}I_{m}^{*}I_{h}}-1+ln\left(\frac{S_{h}I_{m}I_{h}^{*}}{S_{h}^{*}I_{m}^{*}I_{h}}\right)+\frac{S_{m}^{*}}{S_{m}}-1+ln\left(\frac{S_{m}^{*}}{S_{m}}\right)\right]\leq 0.\;\;\;\;\;\;\;\;\;\;\;\;\;\;\;\;\;\;\;\;\;\;\;\;\;\;\;\;\;\;\;\;\;\;\;$$

This implies $\frac{dF}{dt}\leq 0$ holds. And $\frac{dF}{dt}=0$ holds only if $S_{h}=S_{h}^{*},\;V(a,\;t)=V^{*}(a),\;I_{h}=I_{h}^{*},\;R(b,\;t)=R^{*}(b),\;S_{m}=S_{m}^{*},\;L=L^{*},\;E_{m}=E_{m}^{*},\;I_{m}=I_{m}^{*}$, simultaneously. Hence, by the principle of Lyapunov-Lassale’s invariance, the endemic equilibrium is globally asymptotically stable. □
